# Gut microbiota-driven IL-17/PPAR axis mediates epigallocatechin-induced intestinal repair in weaned lambs

**DOI:** 10.1186/s40104-026-01371-5

**Published:** 2026-04-04

**Authors:** Yuwei Zhao, Zhuo Wang, Dingkun Fan, Jixian Zhang, Yan Tu, Qiyu Diao, Kai Cui

**Affiliations:** Beijing Key Laboratory for Dairy Cow Nutrition, Institute of Feed Research, Chinese Academy of Agricultural Sciences, Beijing, 100081 China

**Keywords:** Epigallocatechin, Gut Microbiota, Inflammation, Lamb, Weaning

## Abstract

**Background:**

Early weaning is a key strategy to improve lamb production efficiency; however, it inevitably compromises intestinal barrier integrity and function. This study aimed to investigate the effects of epigallocatechin (EGC) on growth performance and intestinal barrier function in weaned lambs, using metagenomics, metabolomics, and intestinal transcriptomics to elucidate the underlying mechanisms.

**Results:**

Weaning induced oxidative stress, inflammation, and metabolic disruptions in the jejunum. Supplementation with 12.5 mg/kg EGC (LE) significantly improved growth performance, reduced diarrhea incidence (*P* < 0.05), enhanced mucosal antioxidant capacity (*P* < 0.001), and strengthened anti-inflammatory ability (*P* < 0.001). Metagenomic analysis showed that the LE intervention enriched *Ruminococcus* spp. and reduced the abundance of *Slackia*. This microbial shift was associated with elevated luminal concentrations of valeric acid and microbial metabolites derived from EGC. Transcriptomic profiling revealed that the intervention upregulated the PPAR signaling pathway, which supports nutrient metabolism and barrier repair. Concurrently, it attenuated aberrant IL-17 signaling and promoted the restoration of mucosal immune homeostasis, indicating a resolution of excessive inflammatory responses.

**Conclusions:**

Supplementation with 12.5 mg/kg EGC alleviates weaning stress by fostering a beneficial gut microbiota and promoting the production of specific metabolites. These changes reactivate PPAR mediated epithelial repair and dampen pathological immune activation. Low-dose EGC is an effective nutritional strategy to improve intestinal health and growth in weaned ruminants.

**Supplementary Information:**

The online version contains supplementary material available at 10.1186/s40104-026-01371-5.

## Background

Early weaning stress disrupts intestinal barrier function in young animals—including lambs, piglets, and calves—through multiple synergistic pathways [1–3]. It induces intestinal ischemia and hypoxia, resulting in villous atrophy, crypt hyperplasia, and mucosal erosion and bleeding. Moreover, early weaning downregulates the expression of tight junction proteins, such as ZO-1 and Occludin [4, 5], thereby increasing intestinal permeability and triggering bacterial translocation and inflammatory cascade reactions [6–8]. Concurrently, it reduces the secretion of gastric acid, bile, and lysozyme, weakening the gastrointestinal tract’s chemical bactericidal capacity. In addition, early weaning interferes with the expression and glycosylation of mucin-2, further compromising mucosal barrier protection. At the immunological level, early weaning promotes the accumulation of reactive oxygen species (ROS) and lipopolysaccharide (LPS), impairs goblet cell function, reduces secretory IgA (sIgA) production and mucus flow, and inhibits lymphocyte differentiation. Collectively, these effects lead to elevated expression of pro-inflammatory cytokines—including interleukin-1 beta (IL-1β), tumor necrosis factor-alpha (TNF-α), and interferon-gamma (IFN-γ)—and glucocorticoid-mediated immune suppression [9, 10]. Disruption of the microbial barrier allows pathogenic bacteria such as *Clostridium perfringens* to proliferate excessively, releasing endotoxins that further damage epithelial tight junctions [11]. This process activates inflammatory mediators such as IL-8 and TNF-α, perpetuating a vicious cycle of ‘damage–infection–inflammation’, which ultimately results in diarrhea and intestinal mucosal dysfunction in young animals [12].

Epigallocatechin (EGC) is a bioactive plant-derived polyphenol with potent antioxidant, antibacterial, and anti-inflammatory properties. Natural antioxidants have been shown to be effective in scavenging free radicals, thereby alleviating oxidative stress and attenuating the inflammatory response [13, 14]. Beyond its direct effects on the host, the intestinal epithelium interacts with a complex and dynamic microbial ecosystem, the gut microbiota, which profoundly influences host physiology, immune homeostasis, and disease susceptibility through continuous bidirectional signaling [15]. Numerous studies have demonstrated that natural bioactive substances can regulate intestinal microbiota composition and metabolite production, such as short-chain fatty acids (SCFAs), thereby helping to restore intestinal immune balance and barrier integrity [16]. In particular, anaerobic microorganisms in the gut ferment dietary fiber and other carbohydrates to produce SCFAs—including valeric acid, acetate, propionate, and butyrate—which play critical roles in energy metabolism, immune regulation, and the maintenance of intestinal barrier function [17].

It is increasingly recognized that the gut microbiota serves as a key mediator linking dietary nutrients to host biological processes. Given its complex chemical structure and multifunctional properties, EGC may exert beneficial effects by modulating gut microbial communities. Therefore, in this study, we investigated the effects of EGC supplementation administered before and after early weaning on growth performance, immune function, antioxidant capacity, and small intestinal morphology in lambs. Furthermore, we explored host–microbiota interactions to identify key microbial taxa and signaling pathways through which EGC enhances intestinal barrier function, thereby providing new insights into the mechanisms underlying weaning-induced intestinal dysfunction.

## Materials and methods

### Animal, diet, and experimental design

This study was conducted in two experimental batches at Linqing Runlin Livestock Co., Ltd. All experimental procedures were approved by the Animal Ethics Committee of the Feed Research Institute, Chinese Academy of Agricultural Sciences (approval numbers: IFR-CAAS20240429 and IFR-CAAS20240921).

#### Batch 1: early weaning model

A total of 36 twin-born Hu lambs, 21 days of age, were randomly assigned to two groups (*n* = 18 per group): ER1 (maternal suckling) and EW1 (weaned at 28 days of age). The pre-trial period lasted 7 d, followed by a 21-d main trial. Body weight was recorded on d 21, 28, 35, and 42 of age. Diarrhea incidence was monitored daily at 08:00 throughout the trial period. At d 35, 6 lambs from each group were randomly selected and humanely euthanized for sample collection. All lambs were housed in disinfected pens at a controlled ambient temperature of 20–30 °C and had ad libitum access to feed and water. Feed composition is provided in Table S1.

#### Batch 2: EGC intervention trial

A total of 72 weaned Hu lambs (aged 21 d, half male and half female) were randomly assigned to four treatment groups (*n* = 18 per group): ER (maternal suckling), EW (early weaning at 28 days of age), LE (early weaning + 12.5 mg/kg EGC), and HE (early weaning + 50 mg/kg EGC). EGC (≥ 95% purity) was purchased from Xi’an Huamei Technology Co., Ltd. (Xi’an, China). Experimental protocols and procedures followed the same structure as Batch 1. Feed and water were provided ad libitum. On d 35, 6 lambs from each group were randomly selected and euthanized for tissue and intestinal content collection. Feed composition is provided in Table S2.

All pens were cleaned and disinfected prior to the trial. Lambs were individually identified using spray paint. Disinfection was performed every 3 d, and immunization was carried out according to the farm’s standard health management protocol.

### Growth performance

All lambs were weighed weekly to record body weight (BW), and average daily gain (ADG) was calculated for each growth stage. Starting from 7 days of age, feed intake for each pen of lambs was recorded daily to calculate the ADG, average daily feed intake (ADFI), and feed-to-gain ratio (F/G). The incidence of diarrhea was monitored for 14 d after weaning. Diarrhea scoring was based on the physical form of feces, the mental status of the lambs, and the cleanliness of the pen, using a 4-point scoring system [18]. A fecal score ≥ 2 was recorded as one day of diarrhea. If a lamb exhibited diarrhea on any given day, it was recorded as one case of diarrhea. Detailed diarrhea scoring criteria are provided in Table 1. To preclude observational bias, this assessment was performed by trained personnel who remained blinded to the specific dietary allocations of each pen throughout the experimental period.

**Table 1:** Stool scoring criteria

Stool morphology	Score
Normal stool, dry and formed	1
Paste-like stool	2
Semi-liquid stool	3
Liquid stool	4

### Blood chemistry

At 35 days of age, 6 lambs were randomly selected from each group (a total of 24 lambs in Batch 2), and 10 mL of blood was collected from the jugular vein using disposable vacuum blood collection tubes. The blood samples were left to stand at room temperature for 30 min, then centrifuged at 1,430 × *g* to separate the serum. The supernatant was transferred to 2 mL centrifuge tubes and stored at −20 °C. The levels of malondialdehyde (MDA), superoxide dismutase (SOD), catalase (CAT), glutathione peroxidase (GSH-Px), and total antioxidant capacity (T-AOC) in serum were determined using colorimetric assay kits produced by Beijing Jinhaike Yu Biotechnology Development Co., Ltd. (Beijing, China). In addition, the levels of serum immunoglobulin A (IgA), immunoglobulin M (IgM), immunoglobulin G (IgG), and lactate dehydrogenase (LDH) were measured using commercial assay kits. The concentrations of cortisol (COR), tumor necrosis factor-α (TNF-α), interleukin-1β (IL-1β), interleukin-6 (IL-6), and interleukin-10 (IL-10) in serum were detected using enzyme-linked immunosorbent assay (ELISA) kits and measured with a microplate reader.

### Intestinal tissue morphological indicators

After euthanasia, a 2-cm segment from the middle portion of the jejunum was immediately collected. The intestinal contents were rinsed with phosphate-buffered saline (PBS), and the tissue was fixed in 4% paraformaldehyde for subsequent preparation of histological sections. For morphological analysis of intestinal tissue, routine paraffin embedding and hematoxylin–eosin (H&E) staining were performed. Sections were examined under a light microscope. For each sample, five sections were randomly selected, and five representative villi were randomly chosen from each section. Villus height, villus width, and crypt depth were measured using an optical microscope (BX51, Olympus, Tokyo, Japan) equipped with a digital camera (DP25, Olympus) and image analysis software (DP2-BSW, Olympus). The villus height-to-crypt depth ratio was calculated. Additionally, for each section, five intact and well-organized villi were selected to count the number of intraepithelial lymphocytes and goblet cells under the microscope. All intestinal tissue slides were encoded with random identifiers prior to analysis. Key morphometric indices—villus height (VH), crypt depth (CD), and the V/C ratio—were quantified according to the standard definitions outlined in Table S3.

### Mucosal antioxidant and immune markers

Approximately 2 cm segments of the jejunum were placed on a flat ice surface and longitudinally cut open. The digesta was rinsed off using PBS, and the mucosa was washed three times with PBS. The mucosal layer was gently scraped using a clean glass slide, transferred into cryogenic vials, and immediately frozen in liquid nitrogen. The concentrations of IL-1β, IL-4, IL-6, IL-10, IL-17, and TNF-α in the mucosa were determined using ELISA kits. After thawing the mucosal samples at room temperature, an appropriate amount of physiological saline was added for homogenization, followed by centrifugation at 1,500 × g for 10 min, and the supernatant was collected. ELISA kits were purchased from Beijing Jinhaike Yu Biotechnology Development Co., Ltd. (Beijing, China), and the measurements were performed using an ST-360 microplate reader (Shanghai Kehua Bio-Engineering Co., Ltd., Shanghai, China).

The levels of T-AOC, SOD, CAT, GSH-Px, and MDA in the jejunal mucosa were determined using biochemical methods. The corresponding assay kits were purchased from Nanjing Jiancheng Bioengineering Institute (Nanjing, China). After sample preparation, measurements were performed using an L-3180 semi-automatic biochemical analyzer (Shanghai Kehua Bio-Engineering Co., Ltd.).

### Metagenomic sequencing analysis

Jejunal digesta were collected for metagenomic sequencing analysis. Genomic DNA was extracted from stool (0.2 g) using the FastPure Stool DNA Kit (Vazyme, Nanjing, China), quantified (NanoDrop 2000) and quality-checked (1% agarose gel). Libraries (350 bp fragments, Covaris M220) were constructed with NEXTFLEX Rapid DNA-Seq and sequenced on Illumina NovaSeq™ X Plus (NovaSeq X 25B kit) or DNBSEQ-T7. Raw reads were adapter-trimmed and quality-filtered (fastp, length ≥ 50 bp, Q ≥ 20), followed by human genome decontamination (BWA). Clean reads were assembled (MEGAHIT, contigs ≥ 300 bp), ORFs predicted (Prodigal, ≥ 100 bp), and non-redundant genes clustered (CD-HIT, 90% identity/coverage). Gene abundance was quantified (SOAPaligner, 95% identity), with taxonomic (NCBI NR, DIAMOND, E-value < 1e-5) and functional annotation. Differential analysis employed Kruskal–Wallis test at functional/gene levels. The data were analyzed through the free online platform of Majorbio Cloud Platform (https://cloud.majorbio.com/) [19].

### Untargeted metabolomics analyses

Non-targeted metabolomics analysis of lamb jejunal contents was performed at Majorbio Bio-Pharm Technology Co. Ltd. (Shanghai, China). The samples were divided into two portions for LC–MS/MS and GC–MS analyses. LC–MS/MS was conducted on a UHPLC-Q Exactive HF-X system with an ACQUITY HSS T3 column (Waters). GC–MS was performed using a TRACE 1610 GC coupled to an Orbitrap Exploris MS (Thermo Fisher Scientific) with a TG-5SILMS column. Following quality control and identification, the data matrices were combined, duplicate metabolites were removed, and the final dataset was analyzed via the Majorbio platform. The data was pre-processed by retaining metabolites detected in ≥ 80% of samples, estimating values below the lower quantification limit, and normalizing metabolic signatures to the sum. Sample intensities were normalized to reduce preparation and instrument errors. Variables with RSD > 30% were excluded, and log_10_ transformation was applied. Principal Component Analysis (PCA) and OPLS-DA were performed using the R package “ropls” (version 1.6.2) with 7-cycle validation. Significant metabolites (VIP > 1, *P* < 0.05) were identified, and differential metabolites were mapped to KEGG pathways. Enrichment analysis was performed using the “scipy.stats” package to identify relevant biological pathways.

### RNA-seq analysis

Transcriptomic profiling of jejunal tissue was conducted using RNA sequencing (RNA-seq). RNA was extracted with TRIzol^®^ Reagent, with quality verified (OD_260/280_: 1.8–2.2; OD_260/230_ ≥ 2.0; RQN ≥ 6.5; 28S:18S ≥ 1.0; > 1 μg) via NanoDrop 2000. RNA was purified, reverse transcribed, and libraries constructed following Illumina^®^ Stranded mRNA Prep instructions. DEGs were identified using TPM for transcript expression and RSEM for gene quantification [20]. Differential expression analysis was performed with DEGseq [21]. DEGs with |log_2_FC| ≥ 1 and FDR < 0.05 were considered significant. Functional enrichment analysis of GO and KEGG terms was performed using Goatools and Python’s SciPy library, with statistical significance set at *P* < 0.05 following Bonferroni correction. The data were analyzed through the free online platform of Majorbio Cloud Platform (https://cloud.majorbio.com/).

### Statistical analysis

Statistical analyses of ADG, BW, antioxidant indices, and immune markers were conducted using SAS software version 9.4 (SAS Institute, Cary, NC, USA). For Batch 1 (two groups), data were analyzed using Student’s *t*-test. For Batch 2 (four groups), data were analyzed using one-way analysis of variance (ANOVA), followed by Tukey’s multiple comparison test to determine significant differences between groups. Microbial α-diversity between treatment groups was assessed via the Wilcoxon rank-sum test. β-Diversity was evaluated using principal coordinate analysis (PCoA) with Bray–Curtis distance metrics, and group differences were determined by the Adonis (PERMANOVA) test. Microbial taxonomic differences were analyzed with the Wilcoxon rank-sum test. Linear discriminant analysis effect size (LEfSe) was used to identify significantly enriched phyla, genera, and species, with a threshold of LDA score > 2 and *P* < 0.05 considered significant. To ensure the analysis focused on biologically relevant taxa with substantial community representation, a strict pre-filtration step was applied prior to LEfSe analysis. Only taxa with a mean relative abundance greater than 0.1% were retained, thereby excluding low-abundance features and sequencing artifacts. Spearman’s rank correlation was employed for correlation analyses, with significance defined as Spearman’s |*r*| > 0.50 and *P* < 0.05. A *P* < 0.05 was considered statistically significant throughout the study.

## Results

### Weaning causes changes in the levels of intestinal metabolites in lambs

**Fig. 1:**
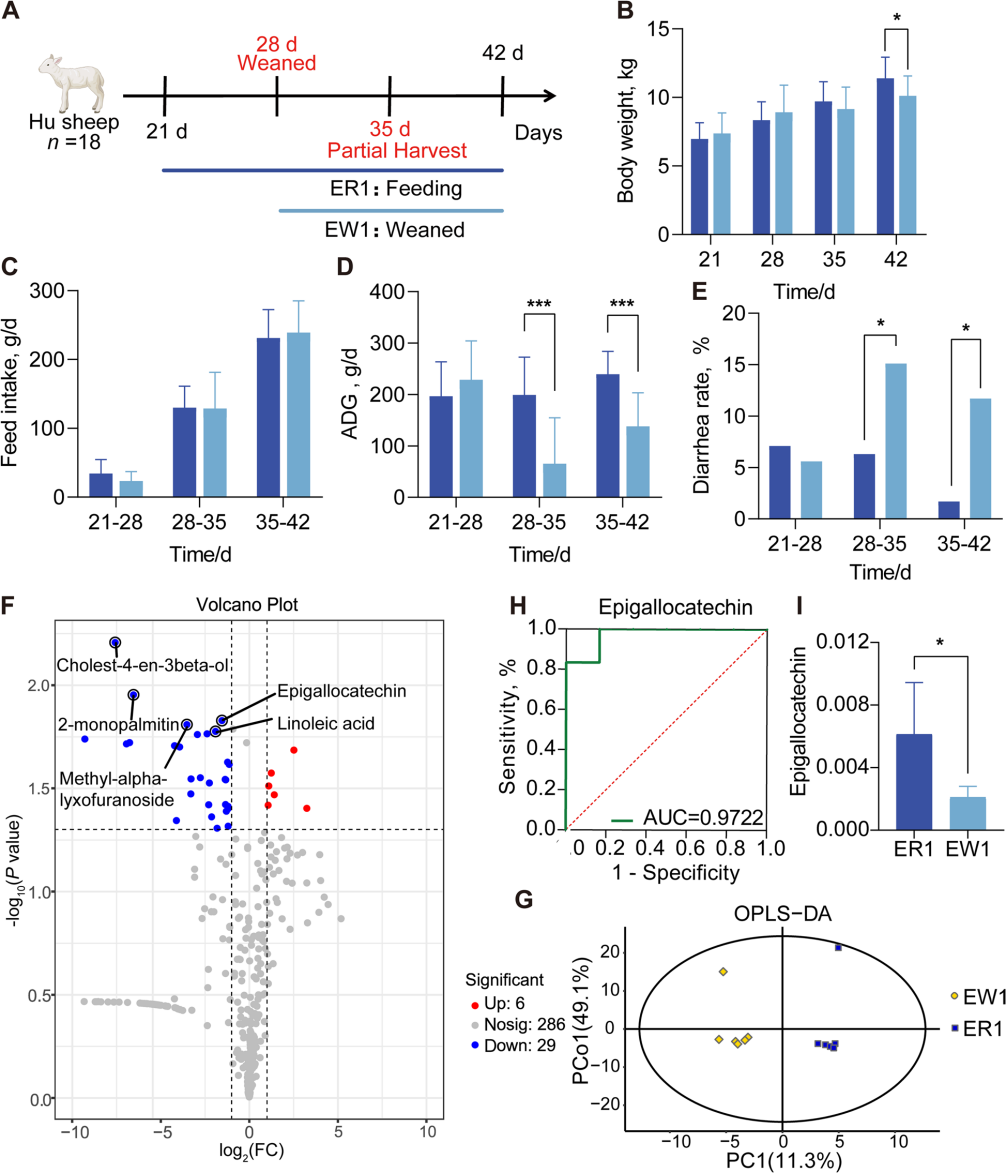
Impact of early weaning on growth performance and jejunal metabolic profile in Hu sheep. **A** Experimental design and timeline. Lambs were divided into the ewe-rearing group (ER1) and the early weaning group (EW1, weaned at 28 days of age). **B** Body weight (BW) changes from d 21 to 42. **C** Feed intake. **D** ADG. **E** Diarrhea rate. **F** Volcano plot of differential metabolites in the jejunum between ER1 and EW1 groups. Blue dots indicate significantly downregulated metabolites, and red dots indicate upregulated metabolites in the EW1 group compared to the ER1 group. Key metabolites, including Epigallocatechin, are labeled. **G** OPLS-DA score plot showing distinct metabolic clustering between the two groups. **H** ROC curve analysis evaluating the sensitivity and specificity of Epigallocatechin as a potential biomarker (AUC = 0.9722). **I** Relative abundance of Epigallocatechin in the jejunal chyme. Data are presented as mean ± SEM. ^*^P < 0.05, ^***^P < 0.001

In the first batch of experiments, it was found that two weeks after weaning, the body weight of the ER1 group was significantly higher than that of the EW1 group (*P* = 0.028; Fig. 1A and B). Weaning stress significantly increased the incidence of diarrhea (*P* < 0.05) and reduced growth performance in lambs (*P* < 0.001) (Fig. 1D and E). Non-targeted metabolomics, combined with OPLS-DA, revealed a clear separation between the ER1 and EW1 groups (Fig. 1G), indicating significant differences in the underlying metabolite profiles. Differential metabolites were screened using the OPLS-DA model, with selection criteria of VIP > 1.0 and *P* < 0.05. A total of 35 differential metabolites were identified; weaning caused significant downregulation of 29 differential metabolites in the jejunal chyme of lambs, while 6 differential metabolites showed significant upregulation (Fig. 1F). Among these, EGC was identified as a key metabolite, showing significant downregulation in the EW1 group (VIP = 1.67, *P* = 0.015) and an AUC of 0.9722, suggesting a strong association with the target phenotype (Fig. 1H and I). Based on these findings, we hypothesized that the depletion of EGC might contribute to intestinal barrier dysfunction, prompting us to investigate its potential protective role.

### EGC improves growth performance and diarrhea rate in weaned lambs

**Fig. 2:**
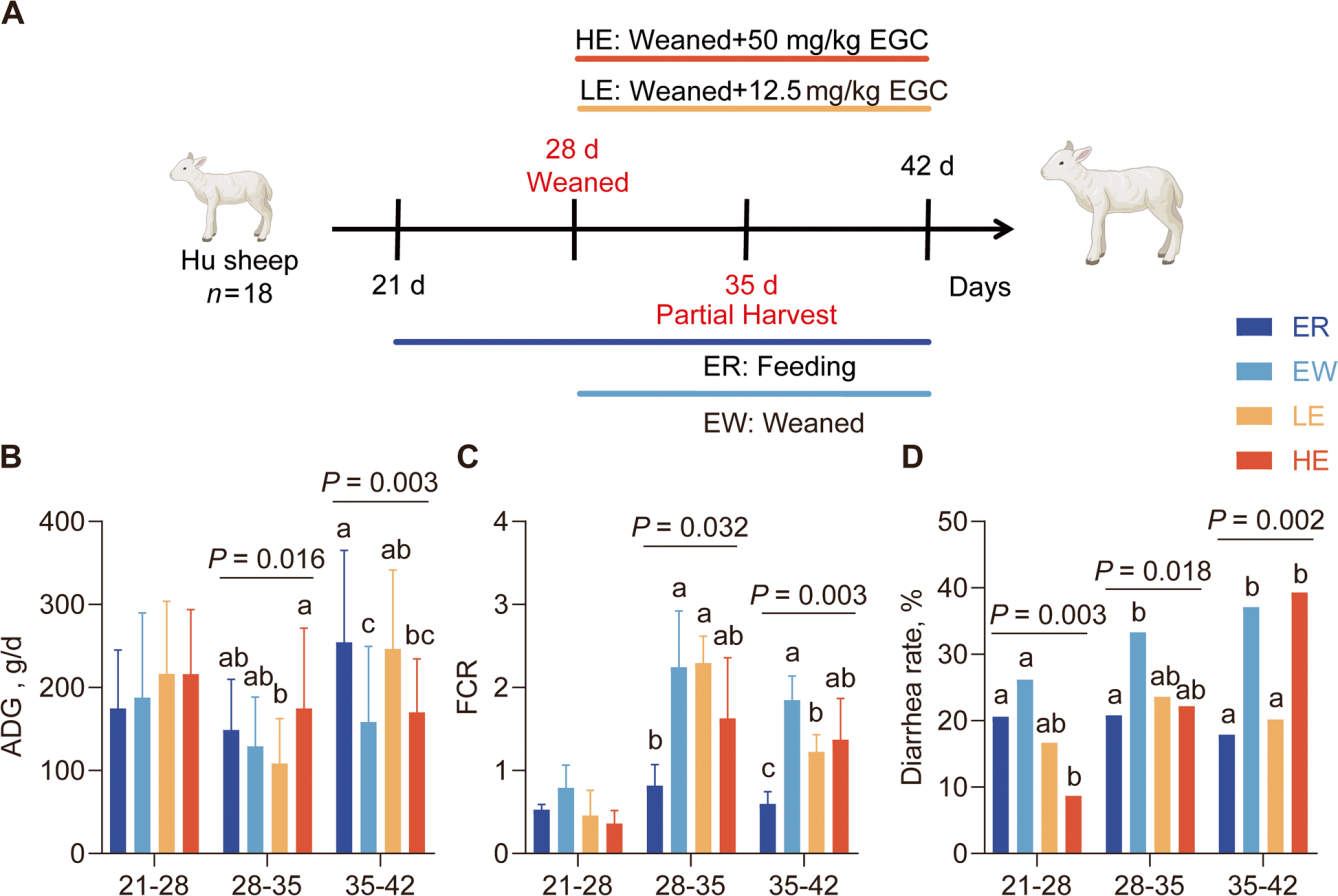
Effects of dietary EGC supplementation on growth performance and diarrhea incidence in weaned lambs. **A** Experimental design and grouping. Lambs were randomly assigned to four groups (*n* = 18): ewe-rearing (ER), early weaning (EW), low-dose EGC (LE, weaned + 12.5 mg/kg EGC), and high-dose EGC (HE, weaned + 50 mg/kg EGC). A partial slaughter was performed at 35 d for sample collection, and the trial concluded at 42 d. **B** Average daily gain (ADG). **C** Feed conversion ratio (FCR). **D** Diarrhea rate. Data are presented as mean ± SEM. Different superscript letters (a, b, c) indicate significant differences among groups (*P* < 0.05)

No significant differences were observed in initial body weight among lambs, nor in body weight among the four groups throughout the experimental period (Additional file 1: Table S4). From d 21 to 35, no significant differences in ADG were observed between the EGC-treated groups and the ER and EW groups (Fig. 2B). During this period, the feed conversion ratio (FCR) of the LE group significantly improved (*P* = 0.003; Fig. 2C). Diarrhea incidence was monitored throughout the experiment. Weaning caused noticeable diarrhea symptoms in the lambs. From d 21 to 28, diarrhea rates in the LE and HE groups decreased, both being lower than those in the ER and EW groups, though the difference for the LE group was not statistically significant. From d 35 to 42, the diarrhea rate in the LE group was significantly lower than that in the EW group (*P* = 0.002; Fig. 2D), returning to levels comparable to the ER group. Conversely, the HE group showed a significant increase in diarrhea incidence relative to the LE group (*P* = 0.002). This observation preliminarily suggests a dose-dependent effect of EGC. In summary, feeding 12.5 mg/kg EGC improved post-weaning lamb growth performance and reduced diarrhea rates.

### Intestinal morphology and barrier function

**Fig. 3:**
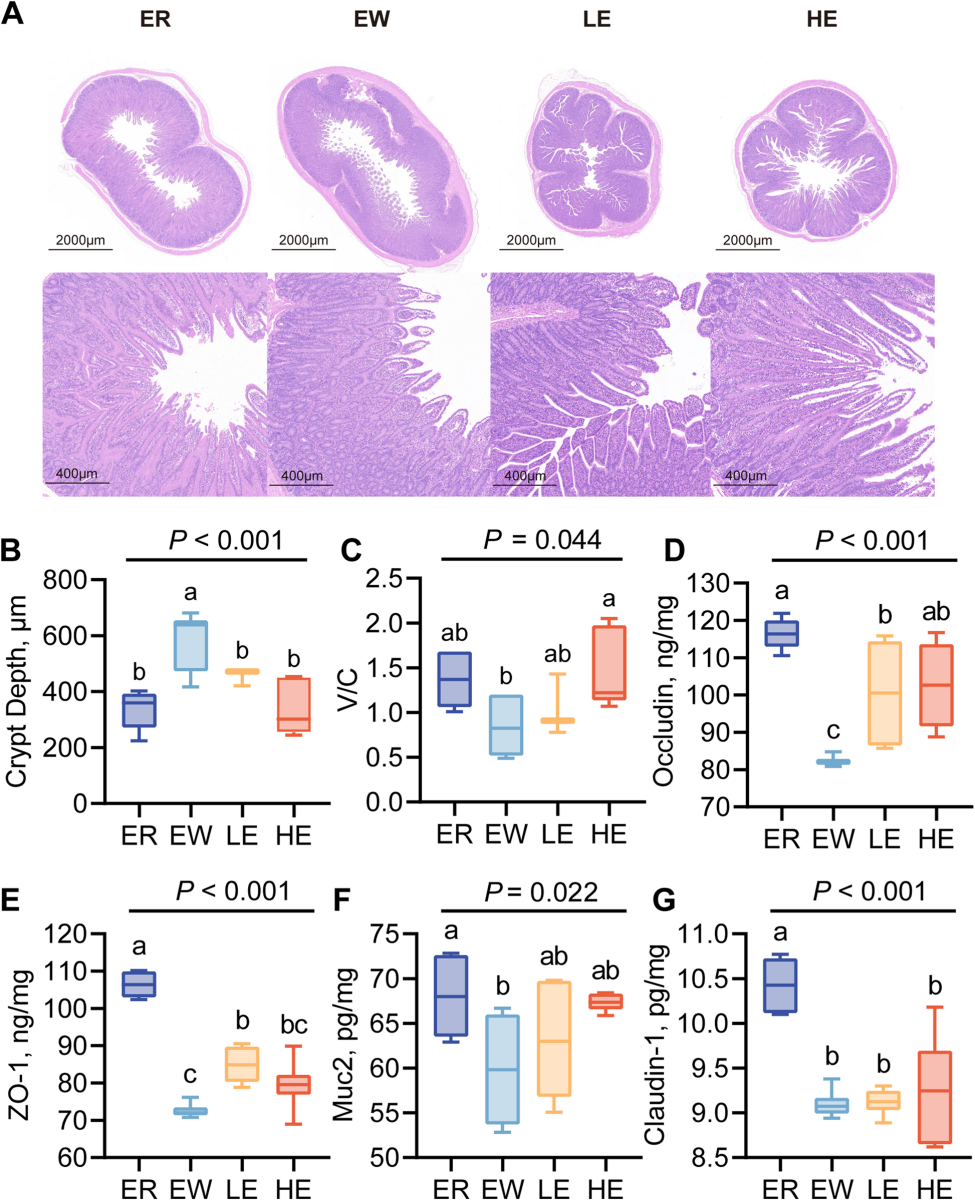
Effects of EGC supplementation on jejunal morphology and barrier function markers in weaned lambs. **A** Representative H&E staining images of jejunal morphology. The top row shows the cross-sections (scale bar = 2000 μm), and the bottom row shows the villi structure details (scale bar = 400 μm). **B** Crypt depth. **C** Ratio of villus height to crypt depth (V/C). **D**–**G** Concentrations of intestinal barrier proteins and mucin in jejunal tissue: (**D**) Occludin, (**E**) ZO-1, (**F**) Muc2, and (**G**) Claudin-1. Data are presented as box plots showing the median, interquartile range, and whiskers (min to max). Different superscript letters (a, b, c) indicate significant differences among groups (*P* < 0.05)

Histological analysis revealed that weaning induced inflammatory cell infiltration and mucosal damage in the lamb jejunum, resulting in a significant increase in crypt depth. However, feeding EGC significantly restored crypt depth and improved the villus-to-crypt ratio (*P* = 0.044; Fig. 3A–C).

Similarly, early weaning significantly reduced the levels of ZO-1 (*P* < 0.001), Claudin-1 (*P* < 0.001), Occludin (*P* < 0.001), and Muc2 (*P* = 0.022) in the jejunum of lambs (Fig. 3D–G). In contrast, compared to the EW group, the LE group showed a marked increase in ZO-1 and Occludin levels (*P* < 0.001), while the HE group exhibited a significant increase in Occludin (*P* < 0.001) (Fig. 3D and E). Although Muc2 levels in the LE and HE groups were numerically elevated, this change did not reach statistical significance (Fig. 3F). These results suggest that, relative to early weaning, continuous maternal feeding preserves superior intestinal barrier function in lambs, and EGC supplementation significantly restores intestinal barrier integrity in the jejunum.

### EGC exerts antioxidant and anti-inflammatory effects at both systemic and jejunal levels

**Fig. 4:**
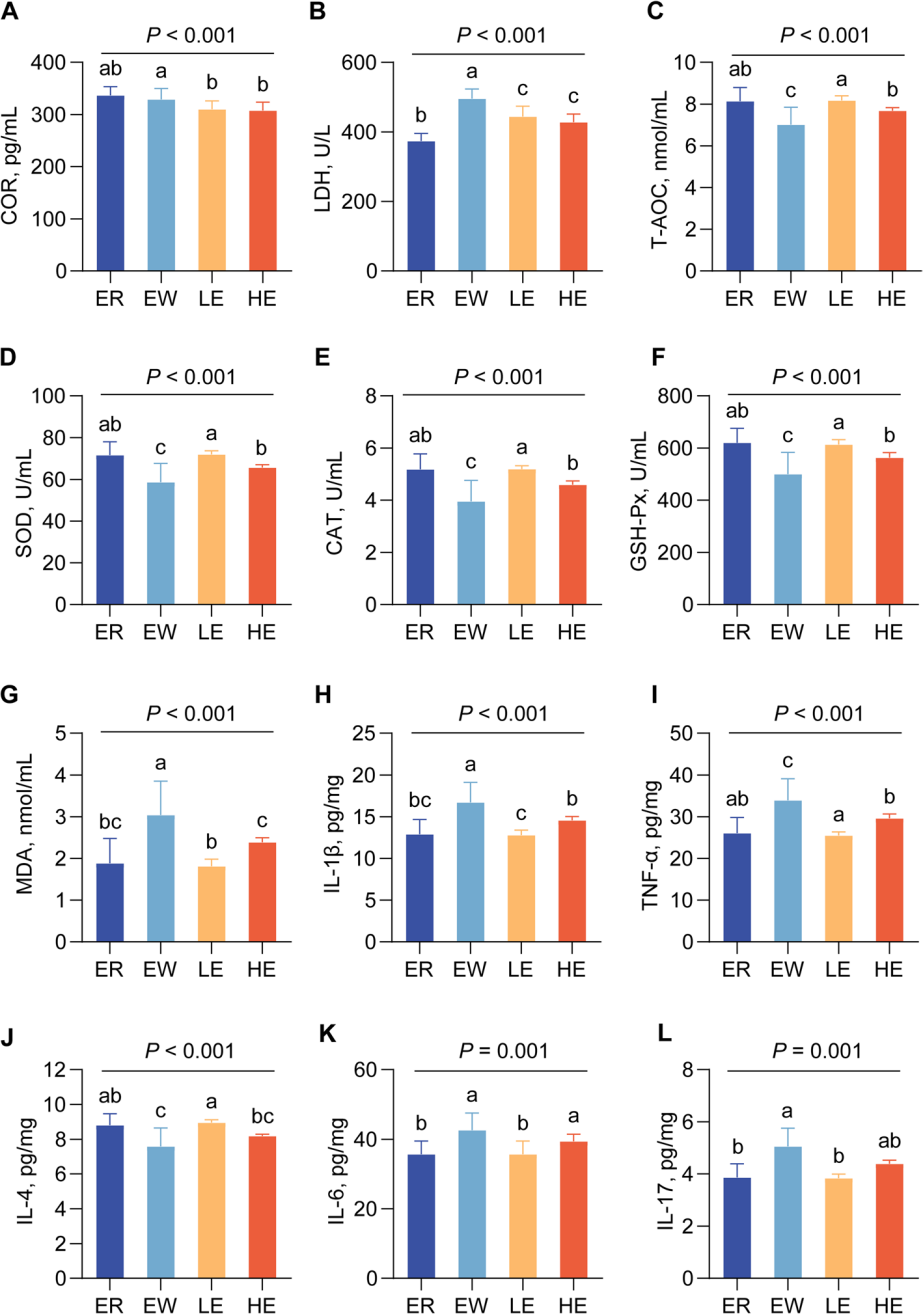
Effects of EGC supplementation on serum stress markers and jejunal mucosal health indices in weaned lambs. **A** and **B** Serum concentrations of stress markers: (**A**) Cortisol (COR) and (**B**) Lactate dehydrogenase (LDH). **C**–**G** Antioxidant and oxidant status in the jejunal mucosa: (**C**) Total antioxidant capacity (T-AOC), (**D**) Superoxide dismutase (SOD), (**E**) Catalase (CAT), (**F**) Glutathione peroxidase (GSH-Px), and (**G**) Malondialdehyde (MDA). **H**–**L** Concentrations of inflammatory cytokines in the jejunal mucosa: (**H**) IL-1β, (**I**) TNF-α, (**J**) IL-4, (**K**) IL-6, and (**L**) IL-17. Data are presented as mean ± SEM. Different superscript letters (a, b, c) indicate significant differences among groups (*P* < 0.05)

COR and LDH are important biomarkers of weaning stress, both of which increase following weaning. Dietary EGC supplementation significantly reduced COR concentrations (*P* < 0.001) and LDH activity (*P* < 0.001), thereby alleviating systemic physiological stress (Fig. 4A and B).

Compared with the ER group, the activities of SOD (*P* = 0.020), CAT (*P* = 0.050), and T-AOC (*P* = 0.048) in the serum of EW lambs were significantly decreased, whereas MDA concentrations were significantly increased (*P* = 0.036). EGC supplementation partially restored systemic antioxidant capacity, although the effect did not reach statistical significance (Additional file 1: Table S5). However, EGC achieved significant improvements in the jejunal mucosa. Consistent with the serum results, weaning decreased intestinal antioxidant capacity, whereas EGC supplementation significantly increased SOD (*P* < 0.001), CAT (*P* < 0.001), and GSH-Px (*P* < 0.001) activities and significantly reduced MDA levels (*P* < 0.001) in the intestinal mucosa. Both EGC-treated groups recovered to levels comparable to those observed during the suckling period (Fig. 4C–G).

Weaning significantly decreased the levels of IgG (*P* = 0.016), IgM (*P* = 0.042), and IL-10 (*P* = 0.028) in lamb serum and significantly increased the levels of TNF-α (*P* = 0.018), IL-1β (*P* = 0.010), and IL-6 (*P* = 0.036) (Additional file 1: Table S6). Dietary EGC supplementation partially restored serum IgG, IgM, and IL-10 levels, but the changes were not statistically significant. Consistent with the jejunal antioxidant findings, weaning significantly increased inflammatory responses in the jejunal mucosa, whereas EGC supplementation significantly reduced TNF-α (*P* < 0.001), IL-1β (*P* < 0.001), IL-6 (*P* = 0.001), and IL-17 (*P* = 0.001) levels and significantly increased IL-4 levels (*P* < 0.001) (Fig. 4H–L). Collectively, these results indicate that dietary EGC significantly alleviated weaning stress by enhancing the antioxidant and anti-inflammatory capacity of the jejunal mucosa. Notably, the protective effects observed in the LE group were superior to those in the HE group.

### EGC modulates the intestinal microbiota composition of weaned lambs

To further explore the gut microbial differences in lambs fed with EGC, we assessed the taxonomic composition of the ER, EW, and LE groups by integrating phenotypic data with microbial profiles. Metagenomic sequencing of jejunal chyme samples was conducted to characterize microbial communities. A total of 2.34 × 10^11^ raw bases and 1.55 × 10^9^ raw reads were obtained (Additional file 1: Table S7). After quality control and host sequence removal, taxonomic annotation revealed a diverse microbial community comprising 4 domains, 11 kingdoms, 207 phyla, 351 classes, 580 orders, 1,068 families, 3,651 genera, and 16,878 species.

**Fig. 5:**
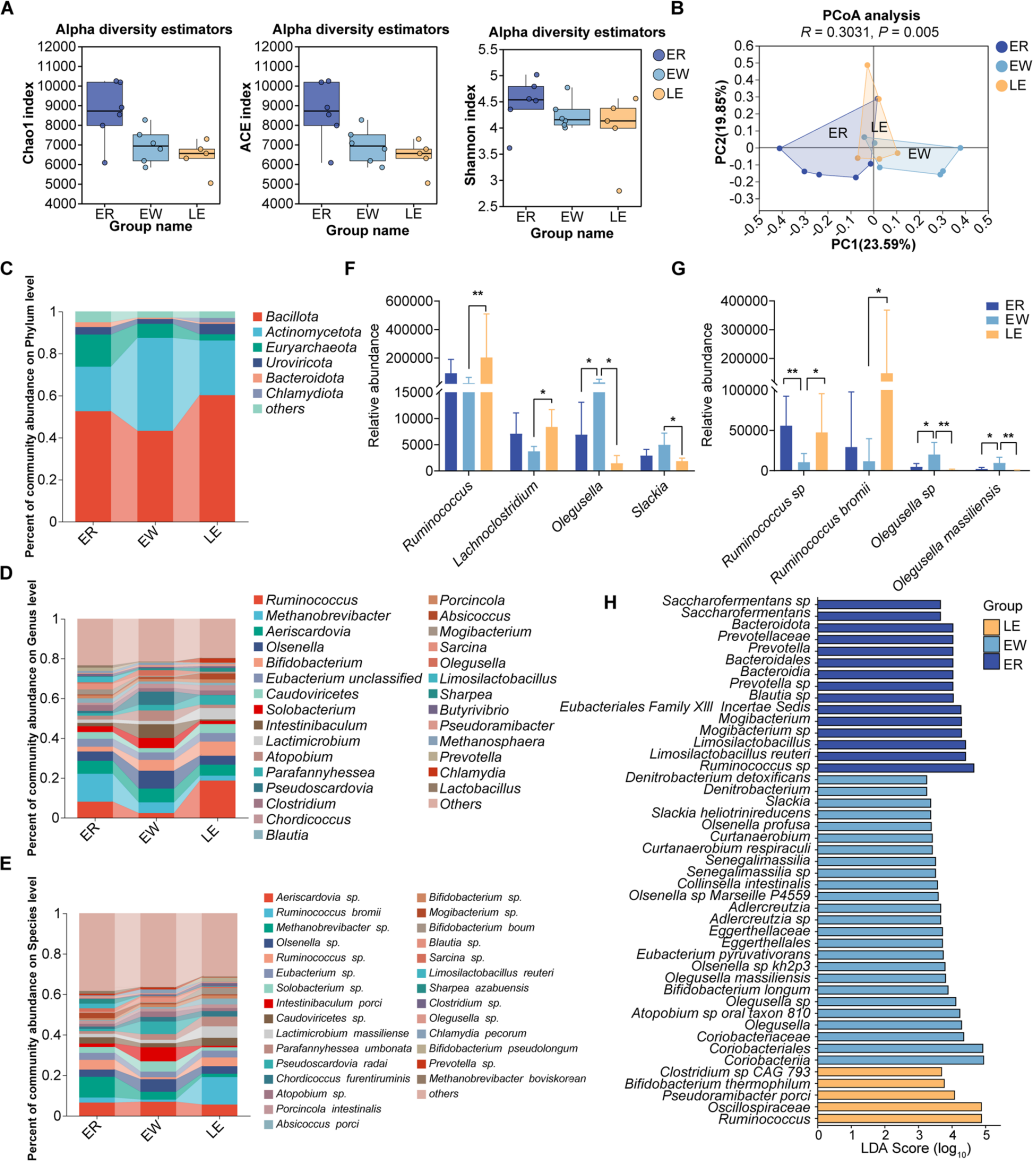
Effects of EGC supplementation on the diversity and composition of the jejunal microbiota in weaned lambs. **A** Alpha diversity indices (Chao1 index, ACE index, and Shannon index). **B** Principal Coordinate Analysis (PCoA) based on Bray–Curtis distance (*R* = 0.3031, *P* = 0.005). **C**–**E** Taxonomic composition of the gut microbiota at the (**C**) phylum, (**D**) genus, and (**E**) species levels. **F** and **G** Relative abundance of differential bacteria at the (**F**) genus and (**G**) species levels. **H** Linear discriminant analysis Effect Size (LEfSe) analysis identifying the biomarkers for each group (LDA score > 2.0). Data in bar charts (**A**, **F**, **G**) are presented as mean ± SEM. ^*^P < 0.05, ^**^P < 0.01

Alpha diversity analysis revealed that weaning did not significantly affect the jejunal microbial diversity (Fig. 5A). However, PCoA based on Bray–Curtis distances demonstrated that both weaning and EGC treatment significantly altered intestinal microbiota β-diversity (*P* = 0.005; Fig. 5B). At the phylum level, weaning decreased the relative abundance of Bacillota and increased the abundance of Actinomycetota, whereas EGC supplementation restored their levels (Fig. 5C). At the genus level, the abundances of *Ruminococcus* and *Lachnoclostridium* were reduced after weaning, whereas genera such as *Olegusella*, *Collinsella*, *Adlercreutzia*, and *Slackia* were enriched. These weaning-induced changes were largely reversed by EGC treatment (Fig. 5D). At the species level, the abundances of *Ruminococcus* sp., *Ruminococcus bromii*, and *Ruminococcus difficilis* were significantly reduced following weaning, whereas *Olegusella* sp. and *Olegusella massiliensis* were significantly increased (Fig. 5E). Notably, these changes were significantly restored in the EGC-treated group (Fig. 5F and G).

LEfSe was applied to identify differential microbial taxa at the phylum, genus, and species levels (Fig. 5H, LDA > 2). LEfSe analysis unveiled distinct genus-level shifts, most notably within the ER group, which was defined by a substantial enrichment of *Limosilactobacillus*, *Prevotella* and *Saccharofermentans*. Distinct from this profile, the EW group favored *Olegusella*, *Adlercreutzia* and *Slackia*, while the LE treatment induced a unique signature dominated by *Ruminococcus*. At the species level, the ER group was defined by a marked abundance of *Limosilactobacillus reuteri*. Diverging from this profile, the EW group exhibited a distinct enrichment of *Slackia heliotrinireducens* and *Olegusella massiliensis*. In contrast, the LE group fostered a unique microbial environment characterized by *Pseudoramibacter porci* and *Bifidobacterium thermophilum*, demonstrating a highly specific reconfiguration of the gut microbiota composition in response to the different interventions.

**Fig. 6:**
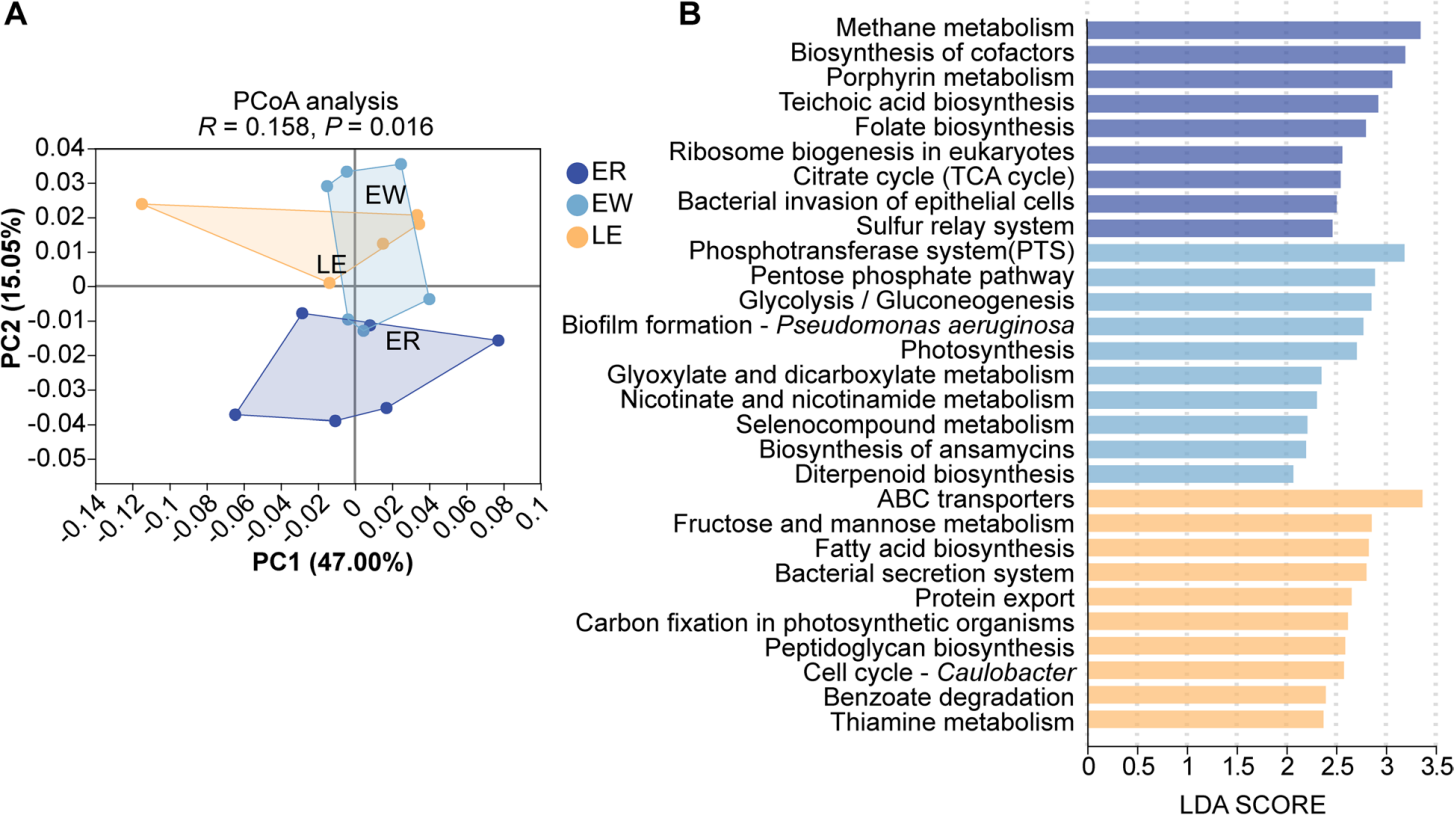
Predicted functional profiles of the jejunal microbiota using KEGG pathway analysis. **A** Principal Coordinate Analysis (PCoA) based on the abundance of KEGG pathways (*R* = 0.158, *P* = 0.016). **B** Linear discriminant analysis Effect Size (LEfSe) analysis identifying the differentially enriched KEGG pathways among groups (LDA score > 2.0)

Subsequently, we analyzed the functional potential of the intestinal microbiota. PCoA diagrams showed clear differences in microbial function (*P* = 0.016; Fig. 6A). LEfSe analysis was performed to identify significantly different microbial functional pathways across the groups. In the EW group, weaning enriched microbial functions associated with low-energy metabolic states, including the phosphotransferase system (PTS), glycolysis/gluconeogenesis, and glyoxylate and dicarboxylate metabolism, which may indicate the proliferation of potentially harmful microorganisms. In contrast, the intestinal microbiota of weaned lambs supplemented with EGC showed enrichment in pathways such as fatty acid biosynthesis, thiamine metabolism, and fructose and mannose metabolism. These functional enhancements suggest improved microbial biosynthesis and metabolic activity, potentially contributing to the maintenance of intestinal homeostasis (Fig. 6B).

### Microbial correlation analysis

**Fig. 7:**
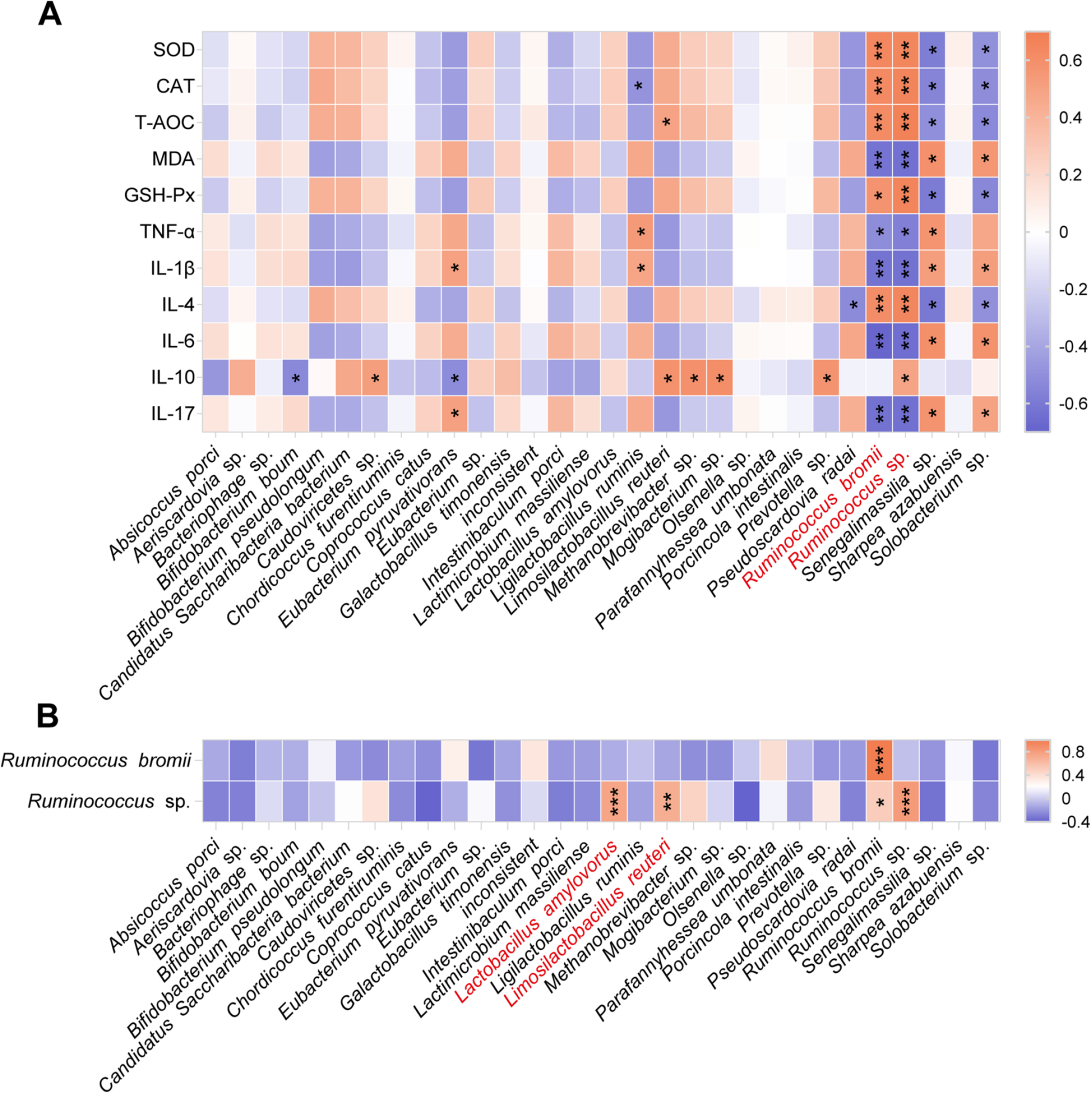
Spearman correlation analysis between the jejunal microbiota and host physiological indices. **A** Heatmap showing the correlations between the top 30 abundant bacterial species and host antioxidant/immune parameters. **B** Focused correlation analysis between key *Ruminococcus* taxa and other bacterial species. Data are presented with significance levels: ^*^P < 0.05, ^**^P < 0.01, ^***^P < 0.001. SOD: superoxide dismutase; CAT: catalase; T-AOC: total antioxidant capacity; MDA: malondialdehyde; GSH-Px: glutathione peroxidase; COR: cortisol; LDH: lactate

To further identify key functional microbial taxa, we performed correlation analyses between specific microbial taxa and jejunal antioxidant and immune indicators. The results indicated that *Bifidobacterium pseudolongum*, *Ruminococcus bromii*, and *Ruminococcus* sp. were positively correlated with intestinal antioxidant capacity, whereas *Collinsella* sp., *Olsenella uli*, *Senegalimassilia* sp*.*, and *Solobacterium* sp. showed negative correlations. Additionally, *Ruminococcus bromii*, *Ruminococcus* sp., *Crassvirales* sp., and *Bifidobacterium pseudolongum* were positively associated with intestinal mucosal immune function, while *Collinsella* sp., *Eubacterium pyruvativorans*, *Olsenella uli*, *Solobacterium* sp*.*, and *Senegalimassilia* sp. showed negative correlations (Fig. 7A).

Given that *Ruminococcus* spp. are primarily involved in dietary fiber degradation, and considering the enrichment of fatty acid biosynthesis pathways in the LE group microbiota, it is speculated that *Ruminococcus bromii* and *Ruminococcus* sp. may interact with certain beneficial bacteria to influence gut microbial homeostasis. Our results also demonstrated significant correlations between *Limosilactobacillus reuteri* and *Lactobacillus amylovorus*, as well as between *Ruminococcus bromii* and *Ruminococcus* sp. (Fig. 7B). Both *Limosilactobacillus reuteri* and *Lactobacillus amylovorus* are recognized probiotics that can prevent intestinal inflammation and contribute to energy homeostasis through metabolite production [22, 23].

### EGC modulates intestinal metabolism in lambs

**Fig. 8:**
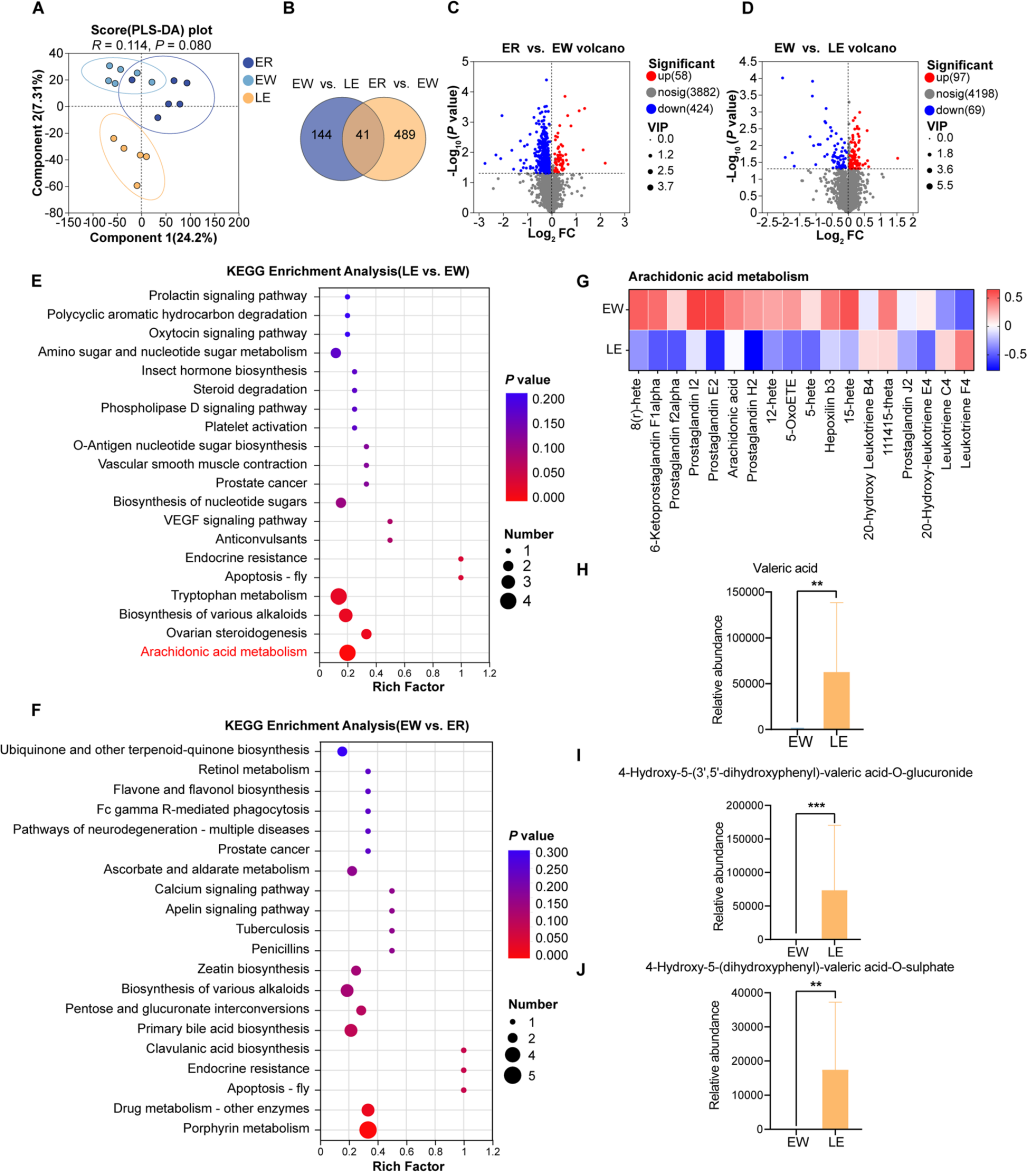
EGC supplementation alters the jejunal metabolomic profile and metabolic pathways in weaned lambs. **A** Score plot of Partial Least Squares Discriminant Analysis (PLS-DA) showing the metabolic trends among groups (*R* = 0.114, *P* = 0.080). **B** Venn diagram showing the common and unique differential metabolites. **C** and **D** Volcano plots for the (**C**) ER vs. EW and (**D**) EW vs. LE comparisons. **E** and **F** KEGG pathway enrichment analysis of differential metabolites for the (**E**) LE vs. EW and (**F**) EW vs. ER comparisons. **G** Heatmap of differential metabolites involved in arachidonic acid metabolism. **H**–**J** Relative abundance of key differential metabolites: (**H**) Valeric acid, (**I**) 4-Hydroxy-5-(3',5'-dihydroxyphenyl)-valeric acid-O-glucuronide, and (**J**) 4-Hydroxy-5-(dihydroxyphenyl)-valeric acid-O-sulfate. Data in bar charts are presented as mean ± SEM. ^*^P < 0.05, ^**^P < 0.01, ^***^P < 0.001

Symbiotic microbial metabolites are recognized as key mediators in host–microbiota interactions. To assess the effects of EGC on the intestinal and microbial metabolism of weaned lambs, we performed untargeted metabolomic analysis of jejunal chyme. Partial least squares discriminant analysis (PLS-DA) revealed distinct metabolic clustering patterns among the three groups, showing a trend of separation (*R* = 0.114, *P* = 0.080; Fig. 8A).

A total of 530 differential metabolites were identified between the EW and ER groups, whereas 185 differential metabolites were identified between the LE and EW groups. Notably, 41 common differential metabolites were shared across both comparisons. Most metabolites were classified as steroids and steroid derivatives (30.00%), prenol lipids (20.00%), and carboxylic acids and their derivatives (13.33%). These distinct metabolic alterations are pivotal in modulating the host's physiological state and functional integrity (Additional file 1: Table S9).

Analysis of the top 30 metabolites ranked by variable importance in projection (VIP) revealed that, compared with the ER group, the EW group had significantly elevated levels of metabolites such as glycochenodeoxycholic acid 3-glucuronide and PS(PGF1alpha/14:0), which are strongly associated with inflammatory processes (Fig. S1A). The accumulation of these metabolites is likely to exacerbate intestinal inflammation. In contrast, EGC supplementation significantly increased the abundance of valeric acid (*P* = 0.002) and EGC-derived microbial metabolites, including 4-hydroxy-5-(3′,5′-dihydroxyphenyl)-valeric acid-O-glucuronide (*P* < 0.001) and 4-hydroxy-5-(dihydroxyphenyl)-valeric acid-O-sulphate (*P* = 0.004) (Fig. 8H–J) [24], and concurrently altered the intestinal microbiota composition (Fig. S1B).

KEGG results showed that weaning led to the enrichment of differential metabolites in pathways such as Porphyrin metabolism, Primary bile acid biosynthesis, and Clavulanic acid biosynthesis. However, after EGC treatment, differential metabolites in the weaned lambs were enriched in pathways including Arachidonic acid metabolism, Tryptophan metabolism, and Ovarian steroidogenesis (Fig. 8F). Notably, the differential abundance (DA) score for the arachidonic acid metabolism pathway was reduced, accompanied by significant decreases in inflammation-associated metabolites such as prostaglandin F_2α_, prostaglandin I_2_, and N-arachidonoyl asparagine (Fig. 8G–H; Fig. S2A and B). These findings suggest an attenuation of inflammatory responses and a potential resolution of metabolic dysregulation.

To elucidate the relationship between microorganisms and metabolites, we conducted correlation analyses. The results revealed significant positive correlations between metabolites (specifically valeric acid and EGC microbial-derived derivatives) and potential beneficial bacteria, such as *Bilifractor porci*, *Lactimicrobium massiliense*, *Ruminococcus bromii*, and *Ruminococcus* sp. At the genus level, positive significant correlations were observed with *Lactimicrobium* and *Ruminococcus*. Conversely, significant negative correlations were found with *Adlercreutzia*, *Atopobium*, *Olsenella*, and *Slackia* (Fig. S3A and B).

Collectively, these findings indicate that EGC supplementation modulates intestinal metabolite composition and mitigates weaning-induced dysbiosis.

### EGC regulated transcriptome

**Fig. 9:**
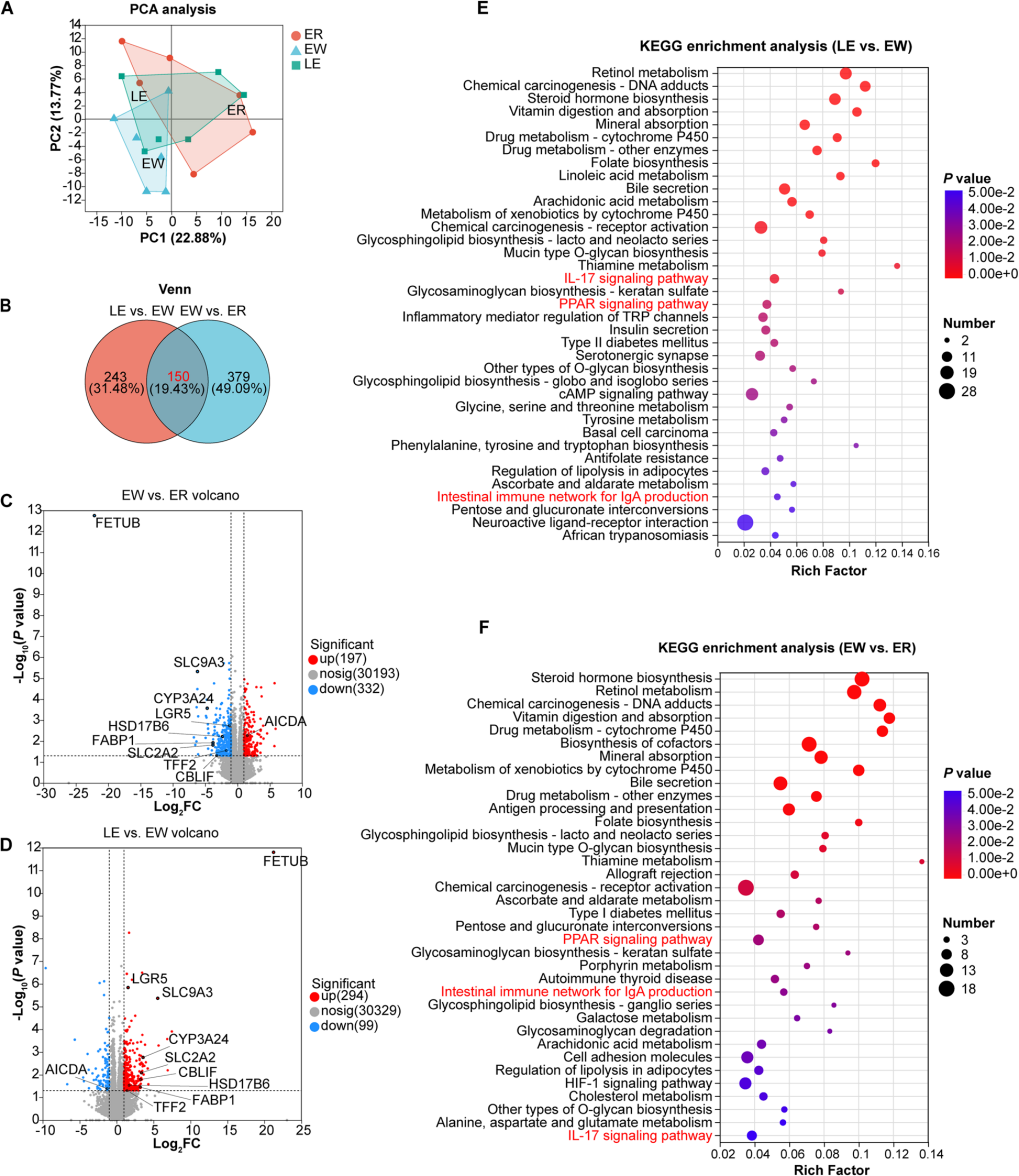
Transcriptomic profiling reveals that EGC supplementation alleviates weaning-induced intestinal barrier damage and inflammation. **A** PCA analysis of the transcriptome. **B** Venn diagram of DEGs. **C** Volcano plot (EW vs. ER). **D** Volcano plot (LE vs. EW). **E** KEGG enrichment analysis (LE vs. EW). **F** KEGG enrichment analysis (EW vs. ER)

To explore the molecular mechanisms underlying the protective effects of EGC, RNA-sequencing was conducted. PCA revealed distinct clustering of the ER, EW, and LE groups, indicating significant transcriptomic alterations induced by weaning and EGC treatment (Fig. 9A).

A total of 529 DEGs (197 upregulated and 332 downregulated, *P* < 0.05, absolute fold-change ≥ 2) were identified in the EW vs. ER comparison, and a total of 393 DEGs (294 upregulated and 99 downregulated, *P* < 0.05, absolute fold-change ≥ 2) were identified in the LE vs. EW comparison (Fig. 9B).

We identified 150 common DEGs shared between the “LE vs. EW” and “EW vs. ER” comparisons. These 150 genes represent the critical transcriptional signature where weaning-induced alterations were effectively reversed by EGC supplementation, serving as potential key drivers for the restoration of intestinal homeostasis.

Key genes related to nutrient absorption (*SLC2A2*, *SLC9A3*, *FABP1*) and epithelial regeneration (*LGR5*, *TFF2*) were significantly upregulated, while the immune activation marker *AICDA* was significantly downregulated in the EGC-treated group (Fig. 9C and D).

The KEGG analysis of the EW and ER groups revealed that the differential genes were enriched in 40 pathways. Notably, these included immune-related pathways (e.g., Antigen Processing and Presentation, Allograft Rejection, Intestinal Immune Network for IgA Production, Cell Adhesion Molecules, IL-17 Signaling) as well as metabolic processes (e.g., PPAR Signaling, Cholesterol Metabolism, Alanine, Aspartate and Glutamate Metabolism) (Fig. 9F). In the LE vs. EW comparison, DEGs were enriched in 37 pathways, notably including IL-17 Signaling, PPAR Signaling, Intestinal Immune Network for IgA Production, and Tyrosine Metabolism (Fig. 9E).

**Fig. 10:**
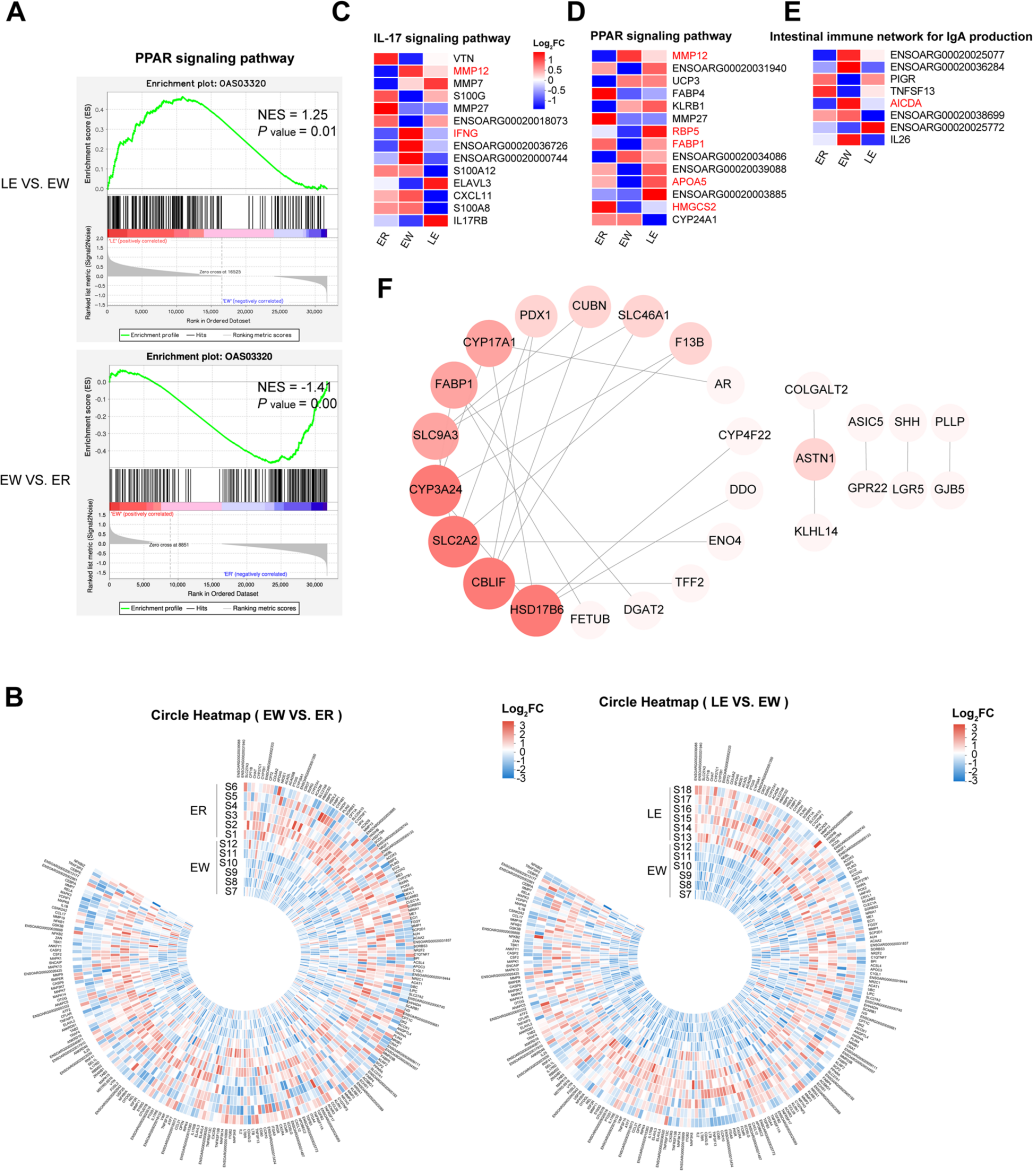
Key signaling pathways and gene interaction networks regulated by EGC. **A** GSEA analysis of the PPAR signaling pathway. **B** Circular heatmaps of DEGs involved in the PPAR signaling pathway. **C** Heatmap of the IL-17 signaling pathway. **D** Heatmap of the PPAR signaling pathway. **E** Heatmap of the intestinal immune network for IgA production. **F** PPI network of hub genes

Given the enrichment of metabolic pathways, we focused on the PPAR signaling pathway, a master regulator of lipid metabolism and energy homeostasis. Gene Set Enrichment Analysis (GSEA) demonstrated that the PPAR signaling pathway was significantly suppressed by weaning stress (Fig. 10A; NES = −1.41, *P* < 0.001) but was notably reactivated by EGC supplementation (Fig. 10A; NES = 1.25, *P* = 0.01). This global reactivation was corroborated by the circular heatmap, which showed a widespread restoration of PPAR-target genes in the LE group (Fig. 10B).

Protein–Protein Interaction (PPI) network analysis of the EGC-reversed genes further identified a dominant “metabolic core” cluster anchored by high-degree hub genes (Fig. 10F). Specifically, the expression of *FABP1*, a key target of PPAR, was significantly upregulated in the LE group. Furthermore, solute carriers essential for nutrient transport, including *SLC2A2* and *SLC9A3*, formed a tight interaction network with *FABP1* and were significantly upregulated upon EGC treatment (Fig. 9C and D). These results suggest that EGC alleviates weaning stress primarily by reactivating PPAR signaling to reconstruct the machinery for nutrient absorption and metabolism.

In addition to metabolic regulation, EGC exerted a profound regulatory effect on mucosal immunity and repair. Heatmap analysis of key immune-related genes revealed that weaning stress triggered an aberrant activation of the IL-17 signaling pathway and IgA production network (Fig. 10C and E). Notably, the expression of *AICDA*, a rate-limiting enzyme for IgA class switching, was sharply elevated in the EW group but was significantly downregulated to baseline levels in the LE group, indicating the resolution of excessive immune stress. We generated heat maps of the co-enriched signaling pathways and found that key genes, such as *MMP12*, *IFNG*, *AICDA*, and other pro-inflammatory genes, were significantly up-regulated in the signaling pathways of the EW group, whereas PPAR-target metabolic genes (e.g., *HMGCS2*, *FABP1*, *APOA5*, *RBP5*) were significantly upregulated in the LE group (Fig. 10C–E).

**Fig. 11:**
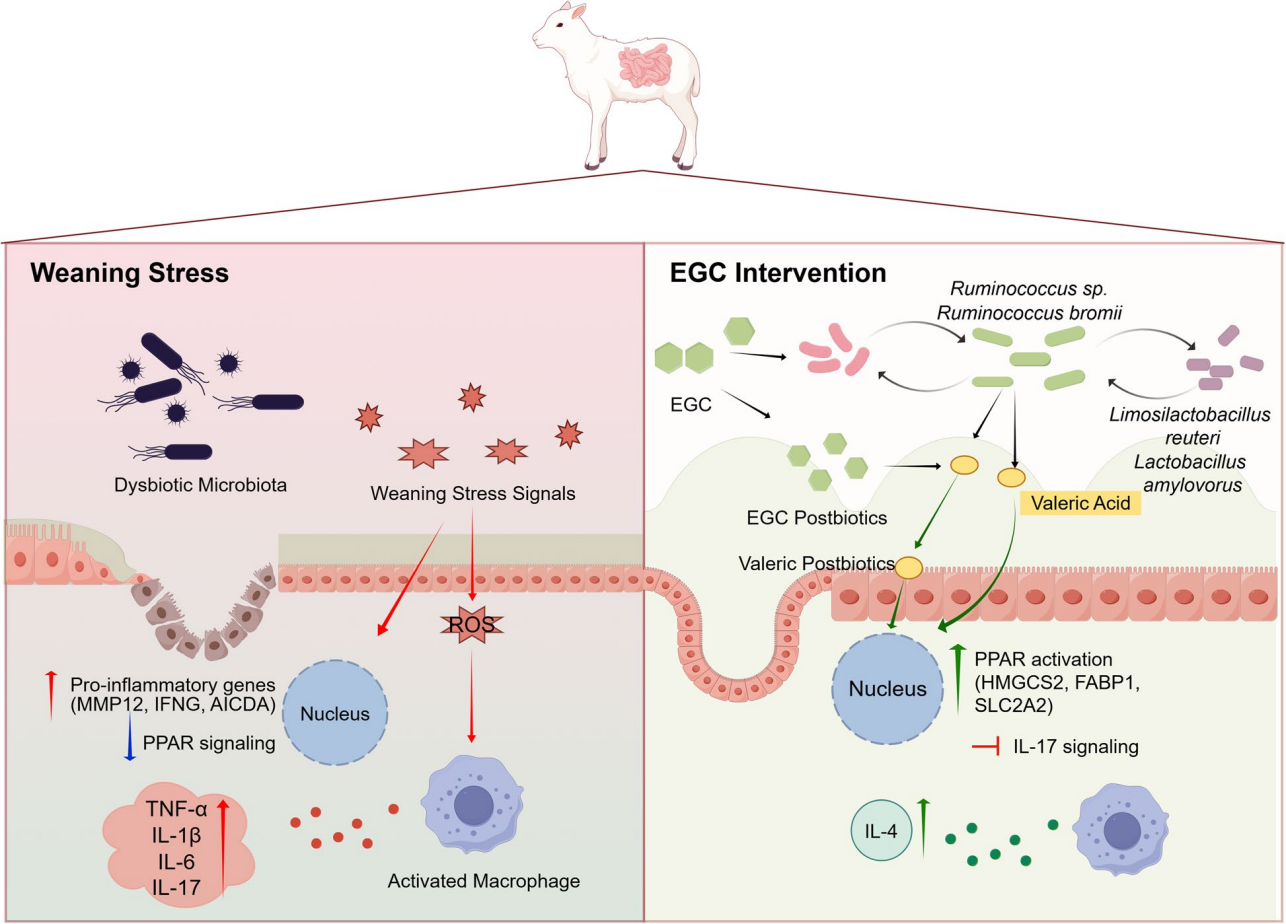
Schematic representation of the potential mechanism by which EGC alleviates weaning stress in lambs via the gut microbiota–metabolite–host signaling axis. (Left Panel) Under weaning stress, the intestinal environment is characterized by dysbiosis, accumulation of oxidative stress signals (ROS), and suppression of PPAR signaling. This redox imbalance triggers the upregulation of pro-inflammatory genes (e.g., *MMP12*, *IFNG*, *AICDA*) and the release of cytokines (IL-17, TNF-α), leading to enterocyte damage, villus atrophy, and barrier dysfunction. (Right Panel) EGC intervention remodels the gut microbiota, specifically promoting the enrichment of *Ruminococcus* spp. and facilitating a cross-feeding network with *Limosilactobacillus reuteri* and *Lactobacillus amylovorus*. This synergistic interaction enhances the production of valeric acid and bioactive EGC postbiotics (ring-fission metabolites). These metabolites act as signaling molecules to activate the PPAR signaling pathway (upregulating *HMGCS2*, *FABP1*, *SLC2A2*) and attenuate the aberrant IL-17 signaling pathway. Collectively, these molecular events mitigate oxidative stress, resolve mucosal inflammation, and restore epithelial barrier integrity. Green arrows indicate activation or promotion; red arrows indicate inhibition or pathological induction. ROS: reactive oxygen species; EGC: epigallocatechin. (Created with figdraw.com)

Concurrently, EGC treatment upregulated the expression of genes associated with mucosal regeneration. In the PPI network (Fig. 10F), we identified a distinct sub-module containing the intestinal stem cell marker *LGR5* and the mucosal repair factor *TFF2*. The restoration of these regenerative factors, combined with the dampening of pro-inflammatory signals, provides a molecular basis for the recovery of villus morphology observed in the histological analysis (Fig. 11).

## Discussion

This study aimed to elucidate the functional relationships within the small intestinal microbiota of weaned lambs supplemented with 12.5 mg/kg EGC, focusing on microbial modulation of oxidative stress and immune responses. Using an integrated metagenomic, metabolomic, and transcriptomic approach, we demonstrate that early weaning induces profound alterations in the intestinal metabolome, directly impacting jejunal physiology. The LE intervention enhanced jejunal antioxidant and anti-inflammatory capacities, significantly improved growth performance, and reduced diarrhea incidence. These effects were accompanied by increased abundance of *Ruminococcus* and elevated luminal levels of valeric acid. Moreover, the intervention reversed the weaning-induced upregulation of pro-inflammatory genes (e.g., *MMP12*, *IFNG*) and increased the expression of metabolic genes (e.g., *HMGCS2*, *FABP1*). Collectively, these findings indicate that low-dose EGC activates the PPAR signaling pathway while modulating the IL-17 signaling pathway and the intestinal immune network for IgA production, thereby improving jejunal epithelial barrier integrity.

Weaning stress represents a critical bottleneck in lamb production. Abrupt weaning, characterized by sudden dietary and environmental shifts, precipitates growth retardation, reduced feed efficiency, and heightened susceptibility to disease, particularly compromising small intestinal function. The most prominent pathological manifestations include villous atrophy and disrupted barrier integrity [25, 26]. Consistent with these established paradigms [27–29], our study confirmed that weaning significantly impaired feed conversion efficiency and intestinal morphology while elevating stress indicators. Notably, the LE group effectively reversed these maladaptive phenotypes. The LE treatment significantly improved feed conversion efficiency, mitigated diarrhea incidence and plasma cortisol levels, and restored intestinal barrier integrity by promoting mucosal repair.

Mechanistically, oxidative stress acts as a primary driver of this post-weaning intestinal dysfunction [30]. While ROS are essential for cellular signaling at physiological levels, the excessive accumulation of ROS during weaning overwhelms endogenous antioxidant defenses. This redox imbalance induces irreversible oxidative damage to nucleic acids, proteins, and lipids, thereby triggering enterocyte apoptosis and necrosis. Such cellular damage contributes to pathological cascades, including mucosal inflammation and barrier failure [31].

In our study, the 12.5 mg/kg EGC intervention significantly enhanced the activity of key mucosal antioxidant enzymes—including CAT, SOD, and GSH-Px—thereby increasing T-AOC in the intestine and reducing MDA concentrations in both the colon and plasma. These effects collectively alleviated weaning-induced oxidative stress. Consistent with these findings, previous studies have demonstrated that catechins exert potent antioxidant properties [32, 33]. Under conditions of severe oxidative stress, ROS can directly activate the NF-κB transcriptional pathway, promoting the production of pro-inflammatory cytokines. Inflammation, in turn, exacerbates ROS-induced pathologies. For instance, activated macrophages release glutathionylated peroxiredoxin-2, which functions as a damage-associated molecular pattern (DAMP) to induce TNF-α expression, further amplifying the inflammatory response [34]. Consistent with previous studies [35], early weaning elevates systemic inflammation in young animals. Here, the LE treatment markedly reduced levels of pro-inflammatory cytokines—including TNF-α, IL-1β, IL-6, and IL-17—in the intestines, while simultaneously increasing anti-inflammatory IL-4 levels. These immunomodulatory effects support the role of low-dose EGC in restoring intestinal barrier function by mitigating oxidative and inflammatory stress.

Crucially, the superior efficacy of the 12.5 mg/kg dose compared to the 50 mg/kg dose suggests a hormetic threshold defined by the physiological immaturity of post-weaning lambs. While the 50 mg/kg dose remains within theoretical safety margins, the immature intestinal landscape appears particularly vulnerable to luminal perturbations. Specifically, the high affinity of excess EGC for digestive enzymes likely inhibited hydrolysis [36], transforming the supplement into a transient antinutritional factor. This localized irritation, evidenced by increased diarrhea in the HE group, compromised the systemic metabolic benefits observed at the lower dosage. To further elucidate the host–microbiota interactions underlying the protective effects observed in the LE group, we performed an integrated analysis combining metagenomics, metabolomics, and transcriptomics. This multi-omics approach enabled the identification of host regulatory targets associated with EGC-induced intestinal homeostasis.

Weaning disrupts intestinal function and weakens the epithelial barrier, increasing the risk of microbial translocation. Under homeostatic conditions, commensal microbes form a biofilm on the mucosal surface that prevents pathogen overgrowth through competitive exclusion and metabolite production [37]. Consistent with our previous hypothesis, the intestinal microbiota represents a key target for modulating gut barrier function. Although the LE group did not exhibit significantly restored overall microbial abundance compared to the EW group, it demonstrated notable shifts in community composition that correlate with the observed phenotypic recovery.

Weaning stress led to the enrichment of Actinomycetota, driven primarily by genera such as *Slackia*, *Olegusella* and *Adlercreutzia*. These taxa, while often associated with metabolic functions, have been functionally linked to compromised barrier integrity and inflammation in specific contexts [38]. Rather than acting as passive biomarkers, the proliferation of these opportunistic pathobionts likely accelerates the erosion of the protective mucus layer, facilitating pathogen contact and fueling local inflammatory responses [39].

Conversely, the LE group was characterized by the specific enrichment of Bacillota (formerly Firmicutes) members, particularly *Ruminococcus* spp., which serve as distinct markers of gut maturation and barrier recovery [40, 41]. In the context of ruminant weaning, the transition to solid feed often suppresses fibrolytic populations; thus, the recovery of these amylolytic taxa suggests that the dietary strategy in the LE group accelerates the establishment of a stable, mature microbiome. Notably, we identified *Ruminococcus bromii*—a keystone species for resistant starch degradation [42]—as a major target of regulation in the LE group. *Ruminococcus bromii* is pivotal for enhancing the intestinal metabolic pool, as its degradative activity drives the production of SCFAs via metabolic cross-feeding [43, 44]. SCFAs are essential for maintaining epithelial hypoxia and upregulating tight junction proteins via activation of the PI3K/AKT signaling pathway [45, 46].

Furthermore, this restoration of key degraders appears to drive broader community synergies. Consistent with the role of *Ruminococcus* in fiber fermentation, our functional analysis revealed an enrichment of fatty acid biosynthesis pathways in the microbiota of the LE group. This metabolic shift likely facilitates synergistic interactions among beneficial bacteria. Spearman correlation analysis revealed that *Ruminococcus* sp. was significantly positively correlated with the probiotic species *Limosilactobacillus reuteri* and *Lactobacillus amylovorus*. Additionally, a strong positive correlation was observed between *Ruminococcus bromii* and *Ruminococcus* sp. Both *Limosilactobacillus reuteri* and *Lactobacillus amylovorus* are recognized for their ability to prevent intestinal inflammation and contribute to energy homeostasis [22, 23]. It is therefore speculated that the LE intervention fosters a synergistic community where *Ruminococcus* mediated fiber degradation creates a niche that supports the proliferation of specific probiotics. This potential metabolic cross feeding network reinforces gut microbial homeostasis, collectively driving the resolution of mucosal injury and inflammation observed in the LE group.

The metabolic landscape of the jejunal chyme revealed that weaning stress precipitates a profound disruption in intestinal homeostasis, characterized by a distinct “pro-inflammatory metabolic shift”. As evidenced by the clear separation in PLS-DA plots, the EW group accumulated stress associated markers, notably PS(PGF1alpha/14:0) and glycochenodeoxycholic acid 3-glucuronide. These metabolites are known to compromise barrier integrity and fuel mucosal irritation, likely acting as biochemical drivers behind the heightened inflammatory status observed in weaned lambs [47, 48].

In contrast, the LE intervention counteracted these dysbiotic signatures through strategic metabolic reprogramming. Central to this restoration was the specific enrichment of valeric acid and microbial metabolites derived from EGC. Mechanistically, these compounds represent the canonical downstream products of the microbial C ring fission pathway, confirming that the gut microbiota actively transforms dietary EGC into bioavailable postbiotics [49, 50]. Bridging taxonomic composition and metabolic output, our correlation analysis empirically validates this functional coupling. The abundance of these beneficial ring fission metabolites showed strong positive correlations with *Ruminococcus bromii* and *Bilifractor porci*, suggesting that the dietary strategy in the LE group creates a nutrient niche favoring these specific metabolizers.

This microbial metabolic restructuring translated directly into physiological benefits, particularly in the regulation of inflammation. KEGG analysis highlighted a significant downregulation of arachidonic acid metabolism in the LE group, accompanied by reduced mediators like prostaglandin F_2α_ and I_2_. Given that arachidonic acid metabolites are potent precursors to the inflammatory cascade [51], their suppression provides a clear biochemical basis for the attenuated inflammation and restored mucosal health. Collectively, these findings indicate that low dose EGC functions as a holobiont modulator, mitigating weaning stress by harmonizing the interplay between microbial metabolism and host immune responses.

To further elucidate the molecular mechanisms underlying the protective effects of EGC against intestinal injury, we performed RNA-seq analysis. The transition from liquid milk to solid feed presents a severe physiological challenge for lambs, involving nutritional deficits, oxidative stress, and mucosal barrier dysfunction [4, 5]. While the antioxidant properties of EGC are established [13, 14, 52], our transcriptomic and histological profiling reveals a more complex mechanism. We demonstrate that EGC alleviates weaning stress by coordinating a systemic metabolic and immunological reconfiguration in the jejunum. Specifically, EGC reverses the weaning-induced transcriptomic signature by simultaneously reactivating PPAR-driven nutrient absorption and resolving excessive mucosal immune activation.

The primary driver of this protective effect appears to be the restoration of metabolic function mediated by the PPAR signaling pathway. Weaning stress typically suppresses genes involved in lipid and energy metabolism, creating an energy deficit in the gut [53, 54]. Our analysis identified the PPAR signaling pathway as the most significantly enriched module among the genes reversed by EGC. Mechanistically, PPAR activation transcriptionally regulates fatty acid uptake and oxidation [55]. Consistent with this, we observed the robust restoration of FABP1 and SLC2A2 in the EGC-treated group. This reactivation suggests that EGC enables the intestinal epithelium to efficiently utilize lipids and glucose, correcting the energy deficit caused by weaning [53, 56]. Additionally, the upregulation of the sodium-hydrogen exchanger SLC9A3 provides a molecular explanation for the reduced diarrhea incidence, as the loss of this transporter is a key factor in weaning-associated secretory diarrhea [57]. These metabolic improvements are likely the fundamental basis for the observed recovery in growth performance.

Parallel to metabolic recovery, EGC exerted a regulatory effect on mucosal immunity, promoting a return to homeostasis. A critical finding was the expression pattern of AICDA, the rate-limiting enzyme for IgA class-switch recombination [58]. While basal IgA production is essential for commensal control, the distinct upregulation of AICDA, coinciding with the dysregulation of the IL-17 signaling pathway in the weaning group, indicates an acute, compensatory inflammatory response to barrier injury [59]. In contrast, EGC treatment significantly downregulated AICDA and dampened the associated pro-inflammatory signaling. This downregulation reflects the resolution of immune stress rather than immunosuppression. By physically repairing the mucosal barrier, EGC reduces bacterial translocation and antigenic load, thereby diminishing the biological necessity for excessive antibody production and pro-inflammatory cytokine release [60]. This shift from active inflammation to homeostasis allows the jejunum to redirect energy from immune defense back to growth [61].

The restoration of metabolic function and immune homeostasis ultimately supports the recovery of the intestinal stem cell niche. Weaning stress is known to arrest stem cell proliferation, leading to the villous atrophy observed in our histological analysis [25, 26, 33]. Our data show that EGC treatment rescued the expression of the stem cell marker *LGR5* and the mucosal repair factor *TFF2*. Since stem cell maintenance requires sufficient energy substrates and low inflammation [26, 62], the PPAR-mediated energy restoration likely creates a favorable microenvironment for LGR5-positive stem cell renewal. This molecular process directly underpins the structural restoration of villus height and crypt architecture.

In conclusion, EGC functions as a dual-action modulator in the weaning intestine. It acts upstream to reignite PPAR-mediated nutrient absorption while simultaneously dampening downstream IL-17 and IgA hyperactivation. This coordinated restoration of metabolic and immune homeostasis offers a compelling mechanistic basis for using EGC to combat weaning stress in ruminants. Long-term studies are warranted to further define how these early-life interventions translate into sustained benefits for growth performance and systemic health in livestock.

## Conclusion

In summary, dietary supplementation with 12.5 mg/kg EGC improved growth performance and alleviated intestinal injury in weaned lambs. The treatment enriched the abundance of *Ruminococcus* spp*.* in the jejunum, which coincided with elevated levels of valeric acid and microbial metabolites derived from EGC. Our analysis showed that these metabolic changes were associated with the activation of the PPAR signaling pathway, which supports epithelial repair. Additionally, the intervention downregulated genes related to IL-17 and IgA production, thereby resolving intestinal inflammation. Therefore, supplementation with 12.5 mg/kg EGC represents an effective method to improve intestinal barrier function and growth performance in ruminants during weaning.

## Supplementary Information

Additional file 1: Table S1. Nutrient content of the starter feed for the Batch 1 (DM basis), %. Table S2. Nutrient content of the starter feed for the Batch 2(DM basis, %). Table S3. Histological scoring criteria. Table S4. Effect of different treatments on lamb body weight at different time points. Table S5. Effects of different treatments on serum antioxidant indexes of lambs. Table S6. Effects of different treatments on serum immune indexes of lambs. Table S7. Summary of sequence data generated from jejunal samples of ER, EW and LE. Table S8. The relative abundances of the top 10 jejunal bacterial phyla, genera and species between ER, EW and LE lambs. Table S9. Common differential metabolites between ER vs. EW and EW vs. LE.

Additional file 2: Fig. S1. Hierarchical clustering heatmaps and VIP scores of the top differential metabolites. Fig. S2. Differential abundance analysis of KEGG metabolic pathways. Fig. S3. Spearman correlation heatmap of gut microbiota and metabolite levels.

## Data Availability

No datasets were generated or analysed during the current study.

## Abbreviations

ADG: Average daily gain
ADFI: Average daily feed intake
AOC: Antioxidant capacity
BW: Body weight
CAT: Catalase
COR: Cortisol
DEGs: Differentially expressed genes
ELISA: Enzyme-linked immunosorbent assay
EGC: Epigallocatechin
FDR: False discovery rate
F/G: Feed-to-gain ratio
GC–MS: Gas chromatography–mass spectrometry
GSH-Px: Glutathione peroxidase
H&E: Hematoxylin–eosin
IFN-γ: Interferon-γ
IgA: Immunoglobulin A
IgG: Immunoglobulin G
IgM: Immunoglobulin M
IL-1β: Interleukin-1β
IL-4: Interleukin-4
IL-6: Interleukin-6
IL-8: Interleukin-8
IL-10: Interleukin-10
IL-17: Interleukin-17
KEGG: Kyoto Encyclopedia of Genes and Genomes
LC–MS/MS: Liquid chromatography–tandem mass spectrometry
LDH: Lactate dehydrogenase
LEfSe: Linear discriminant analysis Effect Size
LPS: Lipopolysaccharide
MDA: Malondialdehyde
Muc2: Mucin 2
OPLS-DA: Orthogonal partial least squares-discriminant analysis
PCA: Principal component analysis
PCoA: Principal coordinate analysis
PBS: Phosphate-buffered saline
PPAR: Peroxisome proliferator-activated receptor
ROS: Reactive oxygen species
RSEM: RNA-Seq by Expectation Maximization
SCFAs: Short-chain fatty acids
SOD: Superoxide dismutase
T-AOC: Total antioxidant capacity
TNF-α: Tumor necrosis factor-α
VIP: Variable importance in projection
ZO-1: Zonula occludens-1
