# Supplementary material for: Gut microbiota-driven IL-17/PPAR axis mediates epigallocatechin-induced intestinal repair in weaned lambs
Source: J Anim Sci Biotechnol. 2026 Apr 4;17:57. doi: 10.1186/s40104-026-01371-5 (PMC13049842; doi:10.1186/s40104-026-01371-5)
Supplement: Supplementary file 2 — Additional file 2: Fig. S1. Hierarchical clustering heatmaps and VIP scores of the top differential metabolites. Fig. S2. Differential abundance analysis of KEGG metabolic pathways. Fig. S3. Spearman correlation heatmap of gut microbiota and metabolite levels. [file 40104_2026_1371_MOESM2_ESM.docx]

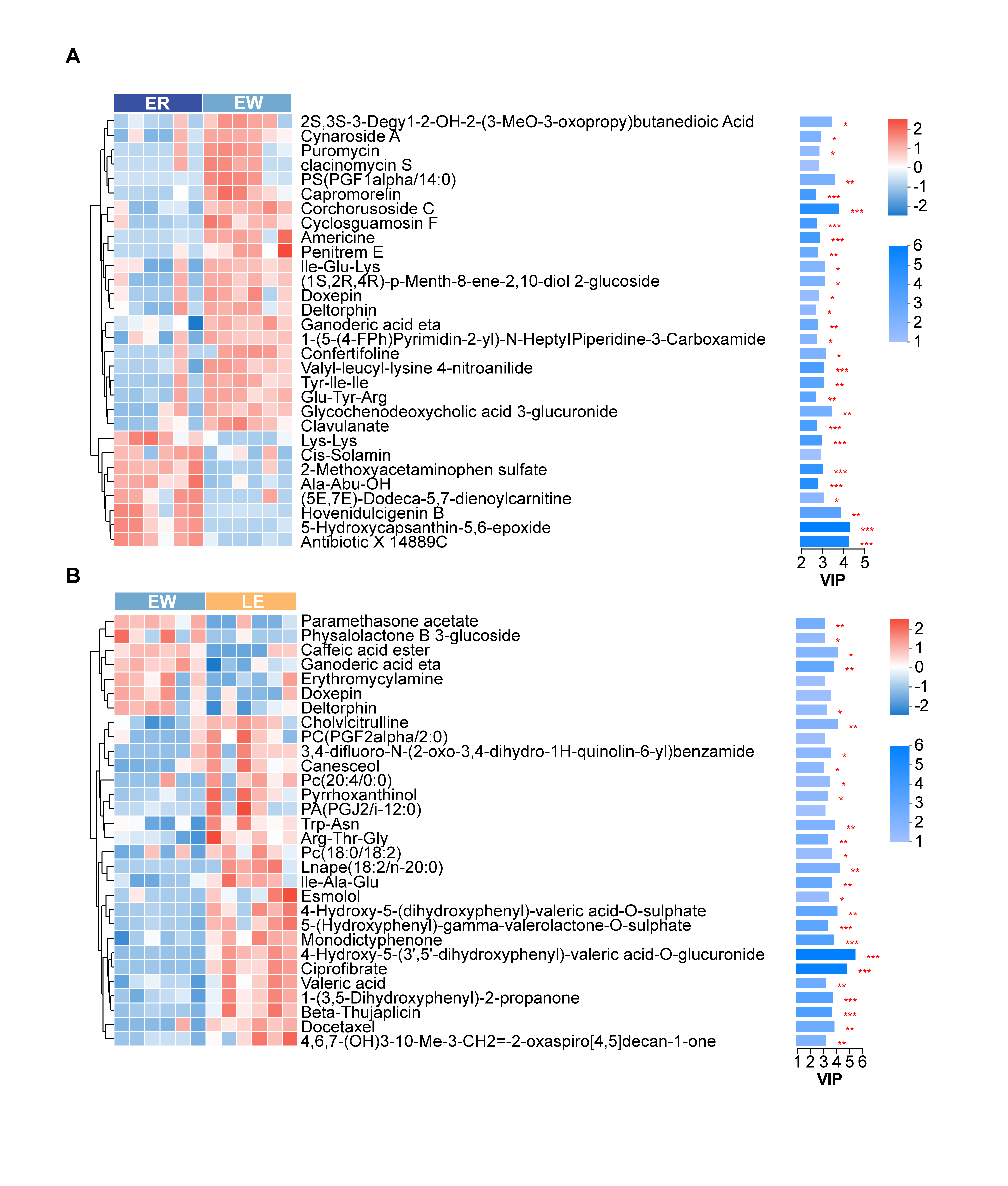


**Figure S1. Hierarchical clustering heatmaps and VIP scores of the top differential metabolites.**

**(A)** Heatmap and VIP scores of differential metabolites identified between the ER and EW groups. **(B)** Heatmap and VIP scores of differential metabolites identified between the EW and LE groups. **P* < 0.05, ***P* < 0.01, ****P* < 0.001.


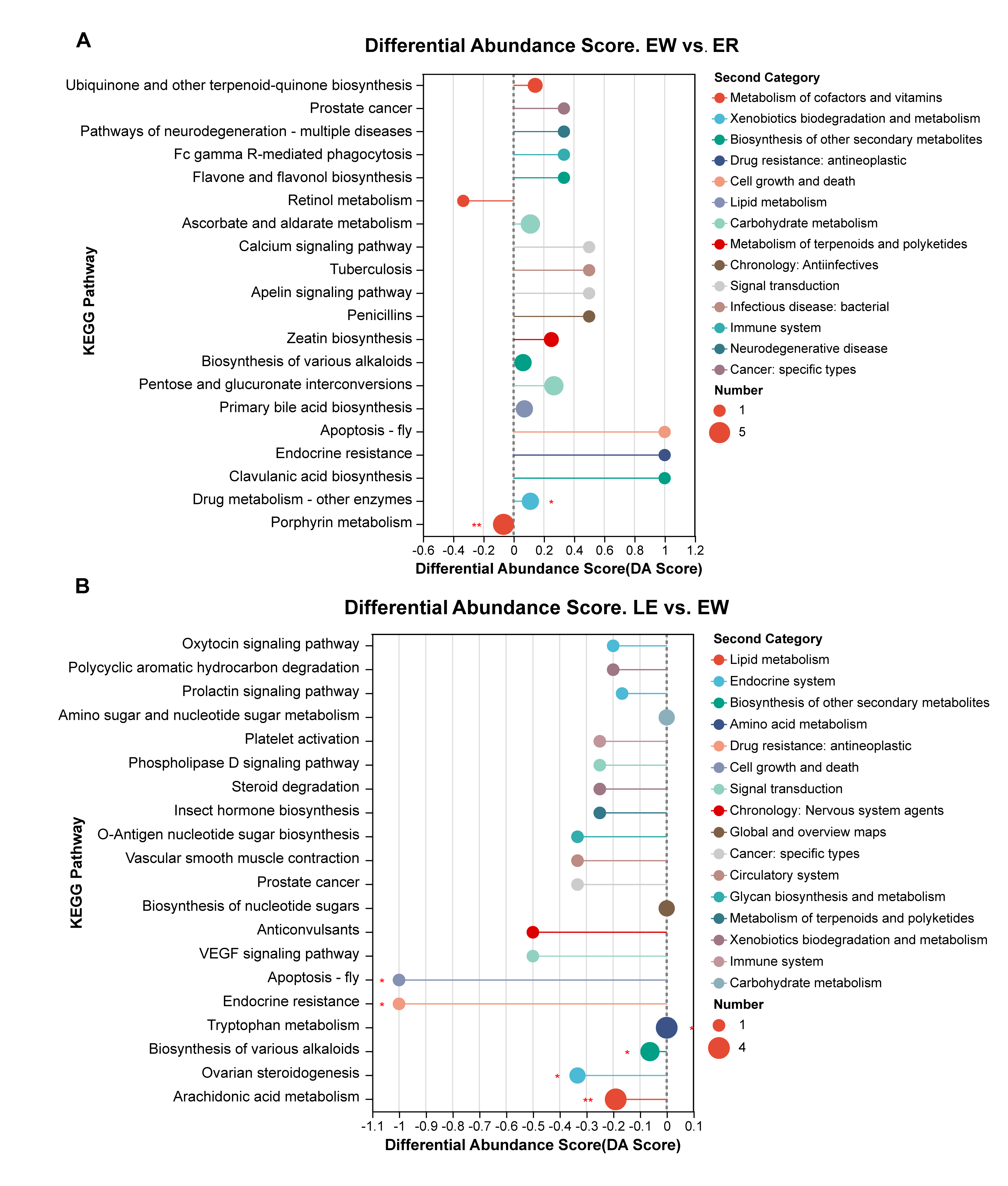


**Figure S2. Differential abundance analysis of KEGG metabolic pathways.**

Lollipop charts displaying the differential abundance (DA) scores of enriched KEGG pathways for the **(A)** EW vs. ER and **(B)** LE vs. EW comparisons. **P* < 0.05, ***P* < 0.01。


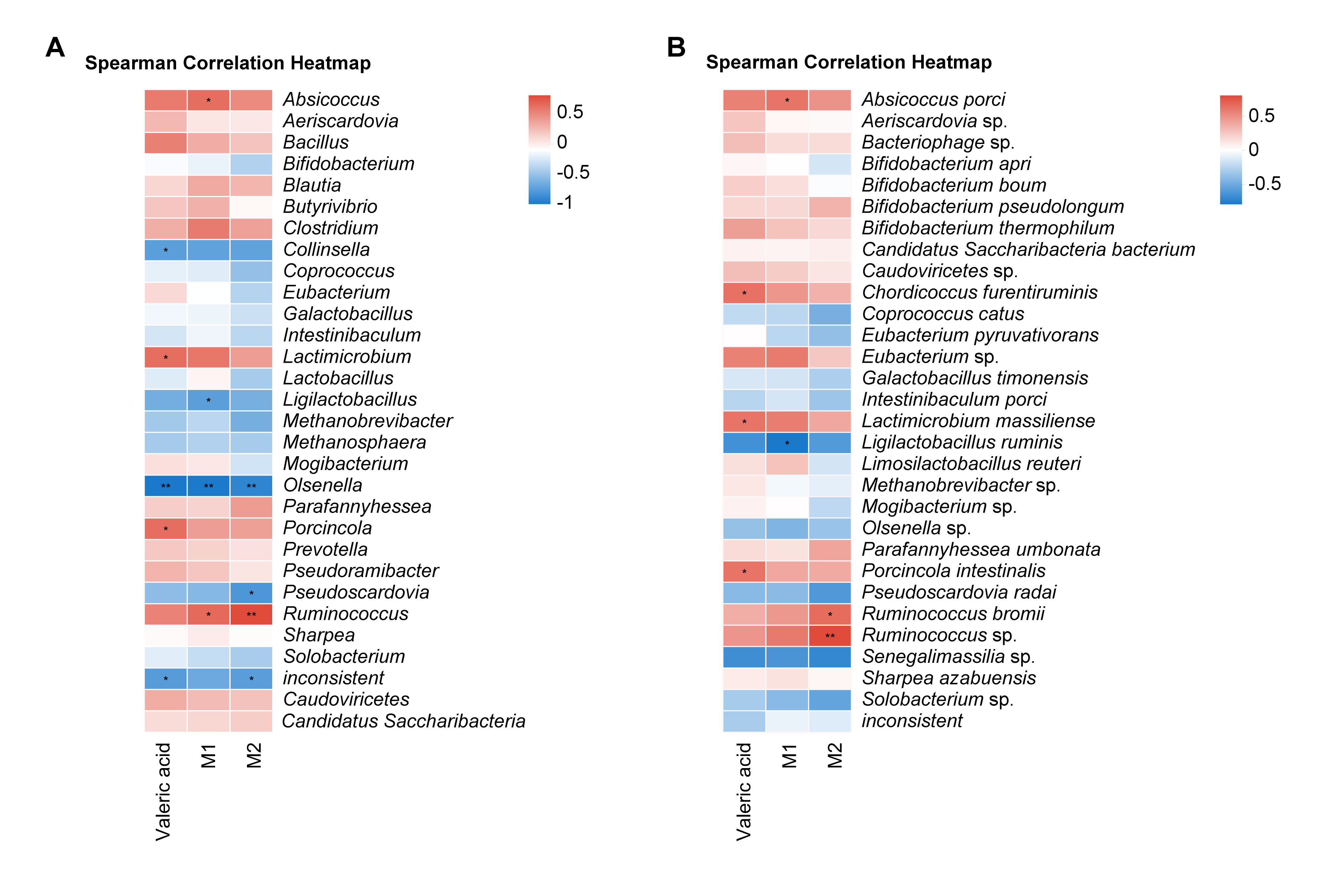


**Figure S3. Spearman Correlation Heatmap of Gut Microbiota and Metabolite Levels.**

**(A)** Spearman Correlation Heatmap of Gut Microbiota at Genus Level with Differential Metabolites. **(B)** Spearman Correlation Heatmap of Gut Microbiota at Species Level with Differential Metabolites. M1: 4-Hydroxy-5-(3',5'-dihydroxyphenyl)-valeric acid-O-glucuronide. M2: 4-Hydroxy-5-(dihydroxyphenyl)-valeric acid-O-sulplate. **P* < 0.05, ***P* < 0.01.
